# Therapeutic Potential of Polyphenols and Other Micronutrients of Marine Origin

**DOI:** 10.3390/md21060323

**Published:** 2023-05-26

**Authors:** Leonel Pereira, João Cotas

**Affiliations:** 1 MARE—Marine and Environmental Sciences Centre/ARNET—Aquatic Research Network, IATV—Institute of Environment, Technology and Life, Department of Life Sciences, University of Coimbra, Calçada Martim de Freitas, 3000-456 Coimbra, Portugal; jcotas@uc.pt; 2 Instituto do Ambiente Tecnologia e Vida, Faculdade de Ciências e Tecnologia, Rua Sílvio Lima, 3030-790 Coimbra, Portugal

**Keywords:** marine polyphenols, therapeutics, antioxidants, anti-inflammatories, health, cardiovascular diseases, diabetes, neurodegenerative diseases, cancer

## Abstract

Polyphenols are compounds found in various plants and foods, known for their antioxidant and anti-inflammatory properties. Recently, researchers have been exploring the therapeutic potential of marine polyphenols and other minor nutrients that are found in algae, fish and crustaceans. These compounds have unique chemical structures and exhibit diverse biological properties, including anti-inflammatory, antioxidant, antimicrobial and antitumor action. Due to these properties, marine polyphenols are being investigated as possible therapeutic agents for the treatment of a wide variety of conditions, such as cardiovascular disease, diabetes, neurodegenerative diseases and cancer. This review focuses on the therapeutic potential of marine polyphenols and their applications in human health, and also, in marine phenolic classes, the extraction methods, purification techniques and future applications of marine phenolic compounds.

## 1. Introduction

The maritime environment encompasses more than 70% of the Earth’s surface and is the world’s biggest ecosystem, with very changeable and hostile physicochemical conditions (low temperature, restricted light availability, high salinity and high pressure). The world’s oceans and seas contain approximately 90% of our planet’s biological biomass, which is dominated by unicellular microbes [1].

The search for natural alternatives for the treatment and prevention of diseases has been increasingly relevant, and marine polyphenols have aroused the interest of researchers in this field. These compounds are bioactive molecules that have antioxidant, anti-inflammatory and antitumor properties, in addition to other beneficial health effects [2]. One of the main sources of marine polyphenols is algae, which contains a diverse range of substances, including flavonoids, phenols and organic acids. Other important sources include fish and crustaceans, which are also rich in marine polyphenols such as catechins and phenolic acids [3].

Marine polyphenols have shown potential for treating and preventing a variety of health conditions. For example, studies indicate that by lowering oxidative stress and inflammation, these substances may help reduce the chance of cardiovascular disease. In addition, marine polyphenols have demonstrated antidiabetic properties, contributing to glycemic control and improving insulin sensitivity [2]. There is also evidence that these compounds may be beneficial for brain health, as they have neuroprotective and anti-inflammatory properties, which may help prevent neurodegenerative diseases such as Alzheimer’s [4]. In addition, marine polyphenols have demonstrated antitumor effects, showing promise in the treatment of several types of cancer. These compounds are believed to help prevent the development of cancer cells, as well as inhibit the growth and proliferation of existing tumors [5].

Due to the therapeutic potential of marine polyphenols, there is a growing interest in the development of nutraceuticals and pharmaceuticals that contain these compounds as active ingredients. However, more studies are required to assess the safety and effectiveness of these compounds in people, as well as to identify the optimal dose for therapeutic use [6].

This review aims to provide a comprehensive understanding of marine organism phenolic compounds and other important compounds, from their origin, highlighting the potential activities as new potential therapeutics to be applied in cardiovascular diseases, diabetes, neurodegenerative diseases and cancer. Furthermore, it will exploit the circular approach: from mechanism of action, safety measures, challenges and extraction/purification methods of the marine-based phenolic compounds.

## 2. Methodology

Data were gathered mostly from internet sources, namely Web of Science, Google Scholar, Science Direct and Scopus, and included research papers, books, chapters, news, websites and reviews. The following subjects were chosen: seaweed, macroalgae, fish, fungi, marine plants, marine and phenolic compounds. In addition, we used a laboratory Mendeley group, which includes article regarding marine phenolic, with all the information gathered from 2019 until now. Furthermore, additional terms such as phlorotannin, bromophenol, terpenoids and flavonoids where also searched. We endeavored to collect as much data as possible with scientific backing for analysis.

However, there are references from before 2019, due to be articles cited in the bibliography analyzed and considered important to cite being the original content cited by the recent bibliography.

## 3. Marine Polyphenols

Marine polyphenols are a group of bioactive compounds that are found in a wide variety of marine organisms, including algae, fish and crustaceans. These compounds are characterized by the presence of multiple hydroxyl groups (-OH) in their molecular structures, which give them antioxidant and anti-inflammatory properties [7]. These compounds have a varied chemical structure and are classified into different groups, such as flavonoids, phenolic acids, tannins, lignans and stilbenes. Flavonoids are one of the most studied classes and include compounds such as catechins, quercetin and rutin, which are commonly found in algae and fish [2].

Marine organisms generate these marine-origin chemicals as a defense strategy against oxidative stress and ultraviolet radiation. Seaweed, for example, is frequently exposed to harsh environmental conditions, and the effects of damage are not visible; as a result, the alga produces a diverse range of metabolites (polyphenols, xanthophylls, tocopherols and polysaccharides) to protect against abiotic and biological factors such as herbivory and mechanical aggression from the sea. Furthermore, marine polyphenols also play an important role in cellular communication and ecological interactions between organisms [8].

Marine polyphenols have aroused the interest of researchers because they have a wide range of health benefits, including anti-inflammatory, antioxidant, antitumor and neuroprotective properties. They have also been investigated as possible therapeutic agents for various conditions such a cardiovascular diseases, diabetes and cancer [9]. Although most studies have fixed their attention on the antioxidant and anti-inflammatory properties of marine polyphenols, recent studies have highlighted the importance of investigating the other mechanisms of action of these compounds, as well as their bioavailability and metabolism in humans [10].

In summary, marine polyphenols are bioactive compounds with promising therapeutic potential, but they are still poorly understood in terms of their properties and effects on human health. Therefore, there is a growing need for additional research to evaluate their safety and efficacy and to develop new therapies based on these compounds [11].

### 3.1. Sources of Marine Polyphenols and Other Micronutrient

Marine polyphenols are found in a variety of natural sources, including algae, fish, crustaceans and mollusks. Below we will detail the main sources of marine polyphenols and the compounds that can be found in each of them [12].

#### 3.1.1. Algae

These bioactive compounds are found in different types of algae, including green (Chlorophyta), brown (Ochrophyta, Phaeophyceae) and red (Rhodophyta) macroalgae [13]. Each type of seaweed has different chemical compositions, with different types and concentrations of polyphenols. They are rich in various types of polyphenols, such as fucoxanthins, phlorotannins and fucoidans [8]. Fucoxanthins are a type of carotenoid found in brown algae and have antioxidant, anti-inflammatory and anti-obesity properties [14]. Phlorotannins are unique phenolic compounds found in brown seaweed that have antioxidant, anti-inflammatory and anti-tumor properties [15]. Fucoidans are sulfated polysaccharides found in brown algae and have antitumor, anticoagulant and anti-inflammatory properties [16].

The polyphenols found in algae are phenolic compounds, which include catechins [17], phlorotannins, fucoidans and fucoxanthins [18]. Catechins are a type of flavonoid that have antioxidant and anti-inflammatory activity, being found mainly in red algae. Phlorotannins are a unique group of polyphenols found in brown seaweed, with antioxidant and anti-inflammatory activity [19]. Fucoidans are sulfated polysaccharides found in brown algae, with anticoagulant, anticancer, anti-inflammatory and immunomodulatory properties [16]. Fucoxanthins are a type of carotenoid unique to brown algae, with antioxidant, anti-inflammatory, anti-obesity and antitumor activity [20].

Seaweed polyphenols have several beneficial properties for human health. In addition to antioxidant and anti-inflammatory activities, these compounds also exhibit antiviral, antifungal and antibacterial activities. Furthermore, studies have shown that seaweed polyphenols exhibit anti-obesity, anti-hypertension, anti-diabetes and anti-cancer activities [21]. Macroalgae polyphenols are also used in cosmetic products such as skin creams and lotions. These compounds have anti-aging, moisturizing and UV-protective properties [22].

Many previous studies have been performed where phenolic compounds were isolated from seaweed and include single phenolic compounds or polyphenols such as flavonoids, phlorotannins, mycosporine-like amino acids (MAAs), bromophenols and terpenoids [23]. The biological action of phenolic compounds is determined by the position of the hydroxyl groups and the number of phenyl rings in the structure [24]. 

Brown algae species contain a large amount of phlorotannins, while green and red algae mainly produce flavonoids, bromophenols, terpenoids and mycosporin amino acids in response to environmental conditions [22]. In the cosmetic industry, phlorotannins enable the activation of hyaluronidase, with antiallergic, anti-wrinkle, anti-aging, skin whitening, photoprotection and improved skin health benefits. Thus, seaweed-derived phenolic compounds and their chemical structures, along with their skin benefits, are extremely useful in the skincare industry [25].

Seaweed-derived phenolic compounds have a wide range of applications, including enzyme inhibition (e.g., tyrosinase inhibition, elastase inhibition, collagenase inhibition, matrix metalloproteinase inhibition in photoprotection, angiotensin-converting enzyme inhibition, 1 (ACE-1), pro-inflammatory cyclooxygenase and lipoxygenase (COX-1, 2 and 5-LOX), as well as inhibition of dipeptidyl peptidase-4 (DPP-4) and inhibition of hydroxymethyl glutaryl coenzyme A reductase (hMGCR)) and antibacterial, antifungal, antioxidant and anti-inflammatory qualities that are appealing when used in makeup and cosmeceutical product formulations [19,22].

It is important to emphasize that the concentrations of polyphenols in seaweed vary according to the species, habitat, environmental conditions, stage of development and extraction method. Therefore, it is important to carry out studies to identify the best sources of polyphenols and the best extraction conditions to ensure obtaining products with a high concentration of bioactive compounds [13]. Among the seaweed species with the greatest potential (see Table 1), the red macroalgae stand out (Rhodophyta): *Neorhodomela larix*, *Rhodomela confervoides*, *Callophycus serratus*, *Tichocarpus crinitus*, *Chondrus crispus*, *Kappaphycus* spp., *Porphyra/Pyropia* spp. and *Symphyocladia latiuscula*; the brown macroalgae (Ochrophyta, Phaeophyceae): *Ecklonia cava*, *E. cava* subsp. *stolonifera*, *E. cava* subsp. *kurome*, *Eisenia bicyclis*, *Ishige okamurae*, *Fucus vesiculosus*, *F. spiralis*, *Gongolaria nodicaulis*, *G. usneoides*, *Laminaria digitata*, *Sargassum muticum*, *S. vulgare*, *S. thunbergii, Lessonia spicata*, *Durvillaea antarctica*, *Vidalia colensoi*, *Padina gymnospora*, *Macrocystis pyrifera*; and the green macroalgae (Chlorophyta): *Caulerpa racemosa, Cladophora socialis*, *Monostroma grevillei*, *Ulva clathrata*, *U. compressa, U. intestinalis, U. linza, U. flexuosa*, *U. australis*, *Capsosiphon fulvescens*, *Chaetomorpha moniligera*.

**Table 1:** Phenolic compounds and other micronutrients from some marine macroalgae and their bioactivities.

Species	Phenolic Compounds and Other Micronutrients	Bioactivities	References
*Callophycus serratus* (R)	Phenolic terpenoids: diterpenes and sesquiterpenes	Antibacterial, antifungal and anticancer	[23,26]
*Capsosiphon fulvescens* (C)	Bromophenols and flavonoids	Antioxidant	[27]
*Caulerpa racemosa* (C) (Figure 1a)	Catechin, epicatechin, epigallocatechin, catechin gallate, epicatechin gallate	Antidiabetic, Antiproliferative, anti-inflammatory and antioxidant	[19]
*Chaetomorpha moniligera* (C)	Bromophenols and flavonoids	Antioxidant	[22,27]
*Chondrus crispus* (R) (Figure 1b)	Isoflavones	Antioxidant, antiproliferative and antidiabetic	[28]
*Cladophora socialis* (C)	Cladophorol	Antibiotic	[29]
*Durvillaea antarctica* (P)	Phlorotannins, tocopherol	Antioxidant	[15,30,31]
*Ecklonia cava* (P)	Polyphenol extract, phlorotannins, cholinesterase, dieckol	Antioxidant, anti-obesity, neuroprotection	[32,33]
*E. cava* subsp. *stolonifera* (P)	Phlorotannins, phlorofucofuroeckol	Anti-inflammatory, antioxidant, anti-hyperlipidemic chemo-preventive	[34,35]
*E. cava* subsp. *kurome* (P)	Phlorotannins	Antibacterial, anti-proliferative, anti-inflammatory and anti-adipogenic	[19,36]
*Eisenia bicyclis* (P)	Phlorotannins, fucofuroeckol-A	Antioxidant, anti-inflammatory and neuroprotective	[23,37]
*Fucus spiralis* (P) (Figure 1c)	Phlorotannins	Antioxidant, photoprotective; anti-enzymatic, anti-inflammatory and cytoprotective	[38,39,40]
*F. vesiculosus* (P) (Figure 1d)	Phlorotannins	Antioxidant, antibacterial and antidiabetic	[41,42]
*Gongolaria nodicaulis* (P) (Figure 1e)	Phlorotannins	Antimicrobial	[23,43]
*G. usneoides* (P) (Figure 1f)	Phlorotannins	Anti-inflammatory, antioxidant, and antimicrobial	[19,44]
*Ishige okamurae* (P)	Phlorotannins	Antioxidant, anti-inflammatory, photoprotective	[45,46]
*Kappaphycus alvarezii* (R) (Figure 1g)	Chlorogenic and salicylic acid	Antioxidant, antimicrobial	[47,48,49]
*Laminaria digitata* (P) (Figure 1h)	Phlorotannins	Antioxidant	[50,51]
*Lessonia spicata* (P)	Phlorotannins	Antioxidant, photoprotective	[52,53]
*Macrocystis pyrifera* (P)	Phlorotannins: phloroeckol and phloroglucinol	Antioxidant and antidiabetic	[54,55]
*Monostroma grevillei* (C)	Polyphenol extract	Antiviral	[23]
*Neorhodomela larix* (R)	Polyphenol extract	Antioxidant	[19,56]
*Padiana boryana* (P)	Ellagic acid and velutin	Antimicrobial and antiprotozoal	[57]
*Padina boergesenii* (P)	Phenolic compounds	Antioxidant and photo-protective	[58]
*Padina gymnospora* (P) (Figure 1i)	Phenolic compounds, flavonoids	Antioxidant, antibacterial	[59,60]
*Polysiphonia morrowii* (R)	5-bromo-3,4-dihydroxybenzaldehyde	Anti-adipogenesis	[61]
*Polycladia myrica* (P)	Phlorotannins	Antioxidant, Antibacterial and photo-protective	[62]
*Rhodomela confervoides* (R)	Bromophenols	Antioxidant, antibacterial, cytotoxic	[63,64,65]
*Sargassum muticum* (P) (Figure 1j)	Phlorotannins, dieckol	Antioxidant, antibacterial, tyrosinase and elastase inhibition	[66,67]
*S. vulgare* (P) (Figure 1k)	Phlorotannins	Antioxidant, antidiabetic, antifungal, pancreatic lipase and anti-inflammatory	[13,68,69]
*S. thunbergii* (P)	Phenolic compounds, phlorotannins	Antioxidant, anti- inflammatory, antibacterial and photoprotective	[70,71,72]
*Symphyocladia latiuscula* (R)	Phenolic compounds, bromophenols	Antioxidant, neuroprotective	[73,74,75]
*Tichocarpus crinitus* (R)	Bromophenols, phenylpropanoids, tichocarpol	Antioxidant, feeding-deterrent activity	[76,77,78]
*Ulva australis* (C)	Phenolic compounds, bromophenols, flavonoids, tannins	Antioxidant, antidiabetic	[19,79]
*U. clathrata* (C) (Figure 1l)	Phenolic compounds, flavonoids	Antioxidant	[80,81,82]
*U. compressa* (C) (Figure 1m)	Phenolic compounds	Antioxidant	[83,84]
*U. flexuosa* (C)	Phlobatanins	Antifungal, antibacterial	[85,86]
*U. intestinalis* (C) (Figure 1n)	Phenolic compounds, flavonoids	Antioxidant, antibacterial	[87,88,89]
*U. lactuca* (C) (Figure 1o)	Ellagic acid and velutin	Antimicrobial	[57]
*U. linza* (C) (Figure 1p)	Phenolic compounds, flavonoids	Antioxidant, anti-inflammatory	[19,81,90]
*U. rigida* (C)	Phenolic compounds	Antifungal, antibacterial, antioxidant and AChE inhibitory capacity	[91,92]
*Vidalia colensoi* (P)	Bromophenols	Antibacterial	[19,23,93]

C—Chlorophyta; R—Rhodophyta; P—Phaeophyceae.

**Figure 1:**
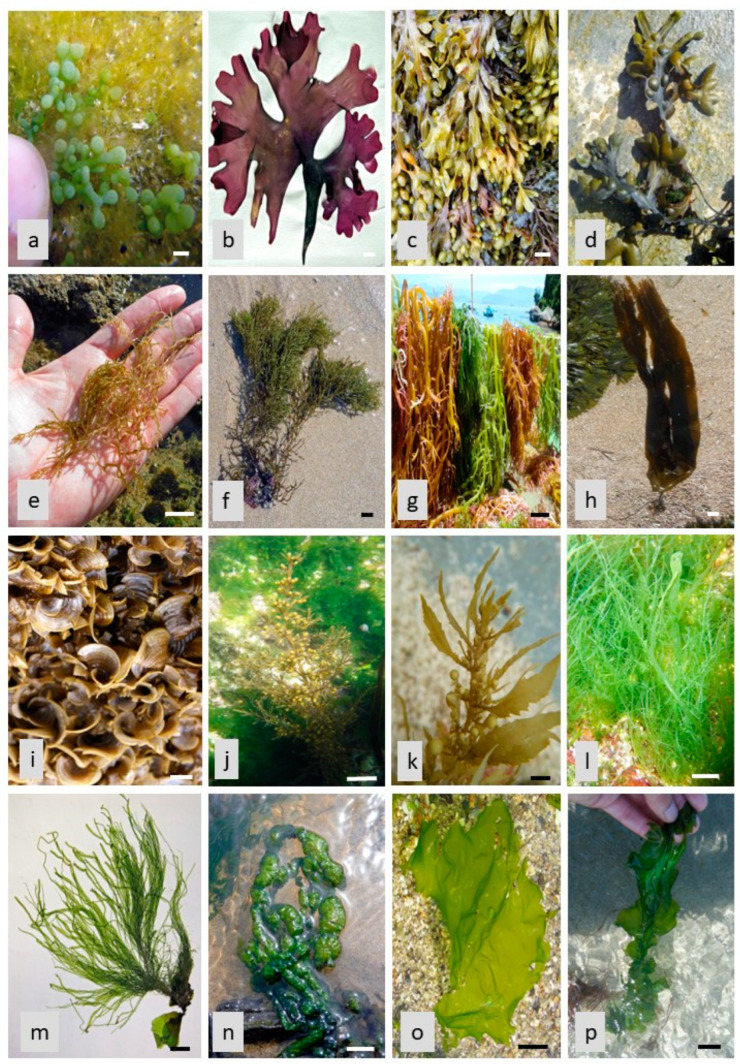
Seaweed species images: (**a**) *Caulerpa racemosa* (C); (**b**) *Chondrus crispus* (R); (**c**) *Fucus spiralis* (P); (**d**) *Fucus vesiculosus* (P); (**e**) *Gongolaria nodicaulis* (P); (**f**) *Gongolaria usneoides* (P); (**g**) *Kappaphycus alvarezii* (R); (**h**) *Laminaria digitata* (P); (**i**) *Padina gymnospora* (P); (**j**) *Sargassum muticum* (P); (**k**) *Sargassum vulgare* (P); (**l**) *Ulva clathrata* (C); (**m**) *Ulva compressa* (C); (**n**) *Ulva intestinalis* (C); (**o**) *Ulva lactuca* (C); (**p**) *Ulva linza* (C); (C) Chlorophyta; (R) Rhodophyta; (P) Phaeophyceae. Scale Bar = 1 cm.

#### 3.1.2. Fish

Fish are also an important source of marine polyphenols and other minor nutrients, particularly fatty fish such as salmon (*Salmo salar*), tuna (*Thunnus orientalis*) and sardines (*Sardina pilchardus*) [94]. Polyphenols found in fish include compounds such as catechins, phenolic acids and carotenoids [95]. Catechins are a type of flavonoid that have antioxidant and anti-inflammatory properties. Phenolic acids are common compounds that are also found in fruits, vegetables and plants that also have antioxidant and anti-inflammatory properties. Carotenoids, such as astaxanthin, are natural pigments found in some types of fish that have antioxidant and anti-inflammatory properties [96].

Curcumin is a natural polyphenol that is found in some fish, such as Tambaqui (*Colossoma macropomum*) [97]. It is responsible for the yellow color of turmeric root, a plant widely used in cooking and traditional medicine [98]. Curcumin has been the subject of many scientific studies due to its antioxidant and anti-inflammatory properties. Curcumin is thought to help prevent or treat a variety of inflammatory conditions, such as arthritis, inflammatory bowel disease and even cardiovascular disease [99]. Additionally, studies suggest that curcumin may help lower blood cholesterol levels. High cholesterol is a major risk factor for heart disease, and curcumin may be helpful in preventing these conditions [100]. Curcumin is considered safe and well tolerated in moderate doses. However, it is important to note that the absorption of curcumin by the body is limited, which can limit its effectiveness in some situations [101].

Catechins are a group of polyphenols with antioxidant and anti-inflammatory properties that are found in many foods, including fish such as tuna and salmon [102]. Catechins are known for their ability to neutralize free radicals, which are unstable molecules naturally produced by the body in response to stress, pollution and other factors. The accumulation of free radicals can lead to cell damage and increase the risk of chronic diseases such as cancer, heart disease and neurodegenerative diseases [103]. Additionally, catechins have anti-inflammatory properties that can help reduce inflammation in the body, which is a natural immune system response to injury and infection, but when persistent can lead to a number of illnesses [104]. Catechins also have anticancer activities, as they can help prevent the growth of cancer cells and inhibit the formation of new blood vessels that feed tumors [105] A study published in the scientific publication “Nutrients” found that eating catechin-rich fish, such as salmon, was associated with a reduced risk of cardiovascular disease [106]. Another study published in “Antioxidants” showed that catechins found in fish can help prevent cellular aging and protect DNA [106]. Although catechins can be found in some fish, most research into their health benefits has been with green tea, which is a rich source of catechins. However, including catechin-rich fish in your diet can be a delicious way to increase your intake of these healthy compounds [107].

Quercetin is a flavonol, a type of flavonoid that is found in many plant foods, including fruits, vegetables and some herbs [108]. Furthermore, quercetin can also be found in some fish such, as salmon and trout. This compound is known for its antioxidant and anti-inflammatory properties and is one of the most studied flavonoids in relation to human health. Quercetin acts as an antioxidant, helping to neutralize free radicals, which are unstable molecules naturally produced by the body that can damage cells and lead to chronic disease [108,109]. Additionally, quercetin has anti-inflammatory properties that can help reduce inflammation in the body, which is a natural immune system response to injury and infection, but which can lead to a host of illnesses when it becomes chronic [110]. Moreover, quercetin may help protect cardiovascular health. It helps to lower LDL cholesterol (“bad cholesterol”) and increase HDL cholesterol (“good cholesterol”), which can help prevent cardiovascular diseases such as heart attacks and strokes. Quercetin also helps lower blood pressure and protects heart cells and blood vessels from damage [111]. Quercetin has also been studied for its potential to prevent and treat cancer. In vivo and in vitro studies have shown that quercetin can help prevent the growth of cancer cells and inhibit the formation of new blood vessels that feed tumors. Additionally, quercetin may help increase the effectiveness of other cancer treatments, such as chemotherapy [112].

Ellagic acid is a naturally occurring phenolic acid that is found in various foods, including fruits, vegetables and some types of fish. Phenolic acids are a type of organic compound that are known for their antioxidant properties and have been associated with a range of health benefits [113] In the case of ellagic acid, research has suggested that it may have anticancer properties and may be beneficial in the prevention and treatment of various types of cancer [114]. Ellagic acid is also believed to have anti-inflammatory and antimicrobial effects, which may further contribute to its potential health benefits [115]. While ellagic acid is most commonly found in fruits and vegetables, such as strawberries, raspberries and pomegranates, it has also been identified in some species of fish. For example, research has shown that ellagic acid can be found in the muscle tissue of salmon and trout (*Oncorhynchus mykiss*) [116]. It is important to note, however, that the amount of ellagic acid present in fish is typically much lower than that found in fruits and vegetables. Therefore, while including fish in one’s diet may provide some small amount of ellagic acid, it is unlikely to have a significant impact on overall ellagic acid intake [117]. Overall, while ellagic acid may be a beneficial compound with potential health benefits, it is important to consider a variety of dietary sources, including fruits, vegetables, fish and other foods, to ensure adequate intake of this and other important nutrients [118].

Fisetin is a natural flavonoid that can be found in a variety of plants and fruits, such as strawberries, grapes, apples, persimmons, onions and cucumbers. It is also present in some fish, including salmon. Research has shown that fisetin possesses powerful antioxidant, anti-inflammatory and neuroprotective properties that may help defend the body against various diseases and health conditions [119]. Antioxidants help to neutralize harmful free radicals in the body, which can damage cells and contribute to the development of chronic diseases such as cancer, heart disease, and Alzheimer’s disease. By reducing oxidative stress, fisetin may help to prevent these conditions from developing [120]. Fisetin has also been found to have cardioprotective effects, meaning it can help protect the heart and cardiovascular system from damage. It may help lower blood pressure and reduce the risk of heart disease by improving blood flow and reducing inflammation in the arteries [121]. In addition to its potential cardiovascular benefits, fisetin has also been studied for its cancer-fighting properties. Some research has shown that fisetin can inhibit the growth and spread of certain types of cancer cells, including prostate, breast and colon cancer cells [122]. Furthermore, fisetin has also been shown to improve cognitive function and memory in some in vivo studies, suggesting that it may have potential benefits for brain health as well [123].

Overall, while more research is needed to fully understand the potential health benefits of fisetin, the current evidence suggests that this natural compound may have a range of health-promoting properties, including antioxidant and anti-inflammatory effects, cardiovascular protection, cancer prevention and potential benefits for brain health [119].

#### 3.1.3. Shellfish

Shellfish, such as shrimps, clams and oysters, are also a source of marine polyphenols and other minor nutrients. The most common compounds found in shellfish are carotenoids such as astaxanthin and zeaxanthin, which have antioxidant and anti-inflammatory properties [124]. These polyphenols are derived from algae and other marine organisms that are consumed by shellfish as part of their diet [3]. One example of a marine polyphenol are the catechins, which are also found in tea, and procyanidins, which are found in various fruits, vegetables and brown seaweeds [17]. These polyphenols are believed to have a range of health benefits, including antioxidant and anti-inflammatory effects [125].

Anther minor nutrient found in shellfish is fucoxanthin, which is a type of carotenoid that is found in brown seaweed. Fucoxanthin has been shown to have antioxidant, anti-inflammatory and anti-obesity properties [126]

#### 3.1.4. Sponges

Despite being a rich source of highly bioactive chemicals [127], there has been little research in the literature on the extraction and identification of polyphenols in sponges. Traditionally, methanol and dichloromethane were utilized for extraction; however, some novel phenolic compounds have been discovered. Bisabolenes are polyphenolic chemicals discovered in sponges that are particularly fascinating. All sponge bisabolenes have a distinct 7S structure, whereas other marine and terrestrial bisabolenes have a 7R structure [127]. (S)-(+)-curcuphenol, a member of this family discovered in sponges, has a variety of biological activities [128].

#### 3.1.5. Marine Fungi

Several Benzaldehyde compounds produced from marine fungus have also sparked interest due to their scavenging characteristics. Wang et al. discovered and characterized chaetopyramin, a scavenging metabolite isolated from the marine fungus *Chaetomium globosum* (Ascomycota) and the red algae *Polysiphonia stricta* (formerly *Polysiphonia urceolata*). Chaetopyramin was synthesized along with known derivatives isotetrahydroauroglaucin and 2-(2′,3′-epoxy-1′,3′-heptadienyl)-6-hydroxy-5-(3-methyl-2-butenyl)benzaldehyde, having DPPH IC_50_ values of 35, 26 and 88 g/mL, respectively [128].

In this case, two additional benzaldehyde derivatives, flavoglaucin and isodihydroauroglaucin, were obtained from the marine fungus *Microsporum* sp. These metabolites, renowned for their DPPH scavenging capacity due to the inclusion of two phenolic hydroxyl groups, demonstrated considerable action, with IC_50_ values in the range of 11.3 and 11.5 g/mL, making them more effective than ascorbic acid (20 g/mL) [128].

The hydroquinone farnesylhydroquinone and its oxidized counterpart, sesquiterpene quinone, were discovered from the marine fungus *Penicillium* sp., and Farnesylhydroquinone (IC_50_ 12.5 M) was shown to be a greater DPPH radical scavenger than ascorbic acid (IC_50_ 22.5 M) [129].

#### 3.1.6. Sea Urchins

The existence of polyhydroxylated naphthoquinone (PHNQ) pigments in sea urchins has long been recognized and investigated [130]. They are concentrated in the shells or gonads, and it has been proposed that they, like other polyphenolic components from edible plants, may be used as antioxidants. Indeed, PHNQs extracted from sea urchin gonads have been demonstrated to be potent antioxidants in lipid peroxidation and food systems [131,132].

However, their use may be hampered by their poor yield and restricted by their brown/orange coloration. The structures of polyhydroxylated naphthoquinone pigments reveal that they are easily reduced and re-oxidized. As a result, their stability is critical for future medical applications. Alternatively, their distinctive quinone structure, along with their structural diversity, may lead to the discovery of novel bioactivities that are more relevant to biological applications [130,133]. 

### 3.2. Phenolic Compounds Metabolomics

There is a natural necessity of extrinsic or intrinsic drivers to make seaweed cellular systems to create naturally and/or enhance/trigger its production from one molecule or a class of chemical to be generated by a specimen in nature or in aquaculture. Primarily (primary metabolites), phenolic compounds (primary and secondary metabolites) are produced naturally and inherently in basic conformations. When seaweed cells are activated in stressful settings, they develop more complex forms [19] As a result, the presence of phenolic chemicals is invariably recognized in cells [19]

Extrinsic factors, on the other hand, activate cellular defensive responses, which can shift the molecular mechanism to produce greater quantities and a wider range of conformations of a specific compound class, particularly when it is a defensive compound synthesized to protect against external attacks [134,135].

If the drivers of seaweed compound production are fully understood, the exploitation of phenolic compounds and their bioactivity can be moved into kinetic models, providing more exploitation safety and information on how to explore phenolic compounds efficiently with lower costs and higher quality [136,137]. Thus, the cultivation of the marine organism under controlled conditions can be a feasible system to produce and obtain a natural phenolic compound that can be applied commercially. One of the examples is the Dieckol from the brown seaweed *Ecklonia radiata*, which is already applied in cardiovascular therapeutics [19].

## 4. Structure and Properties of Marine Polyphenols

The basic structure of marine polyphenols consists of multiple phenolic rings linked together by various chemical bonds. These rings can be modified with other chemical groups, such as sugars or sulfates, which can further influence their properties [138].

One of the unique features of marine polyphenols is their ability to form complex aggregates or “tannins” through intermolecular interactions such as hydrogen bonding and hydrophobic interactions. These tannins can have different physical and chemical properties compared to their monomeric counterparts, including increased solubility and stability [139].

Another important property of marine polyphenols is their potential to be used as natural food preservatives. Some marine polyphenols, such as the phlorotannins found in brown seaweed, have been shown to inhibit the growth of various bacteria and fungi, which can help to extend the shelf life of food products [15].

Because of their structural variety and unpredictability, phenolic compounds from marine creatures are significantly less researched than those from terrestrial sources. However, their biological significance and prospective features make them an appealing category deserving of more scientific investigation. The utilization of effective extraction and, in certain circumstances, purifying processes can provide new bio-actives valuable for food, nutraceutical, cosmeceutical and pharmaceutical applications. The bioactivity of marine phenolics is due to their enzyme inhibitory action as well as antibacterial, antiviral, anticancer, antidiabetic, antioxidant or anti-inflammatory properties [3]. The marine ecosystem can be exploited by aquaculture techniques, causing less impact in terrestrial ecosystem.

### 4.1. Some Phenolic Compound Structures and Bioactivities

#### 4.1.1. Phenolic Acids (PAs)

**Figure 2:**
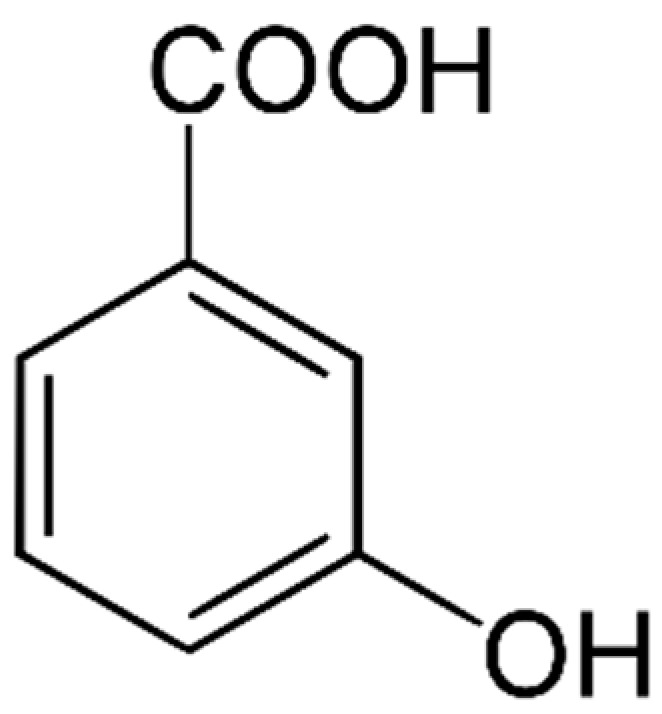
Hydroxybenzoic acid (HBA).

**Figure 3:**
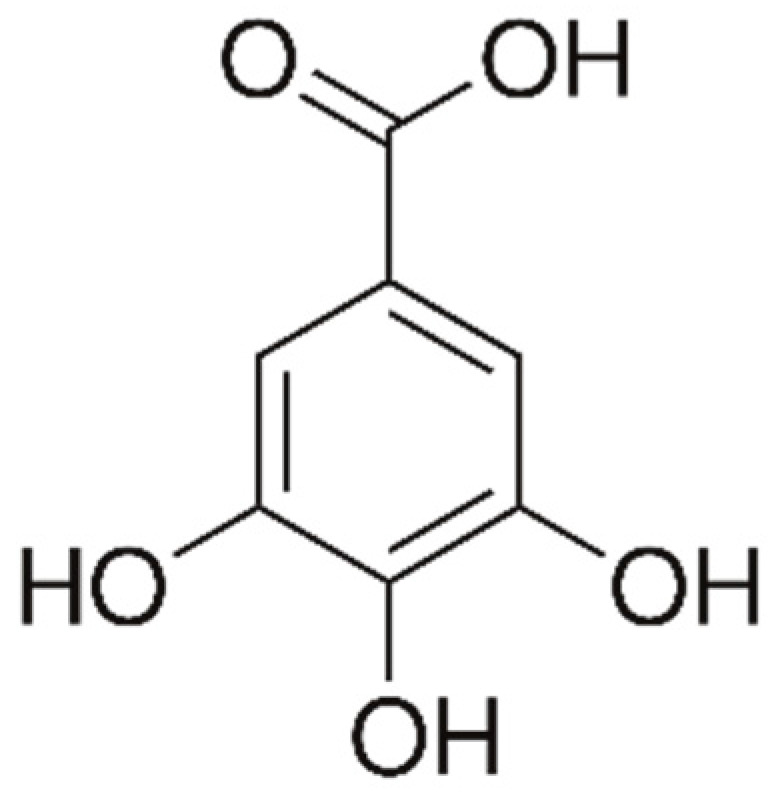
Gallic acid.

**Figure 4:**
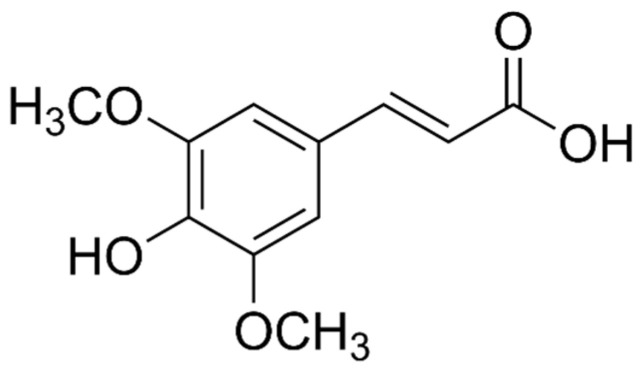
Protocatechuic acid.

**Figure 5:**
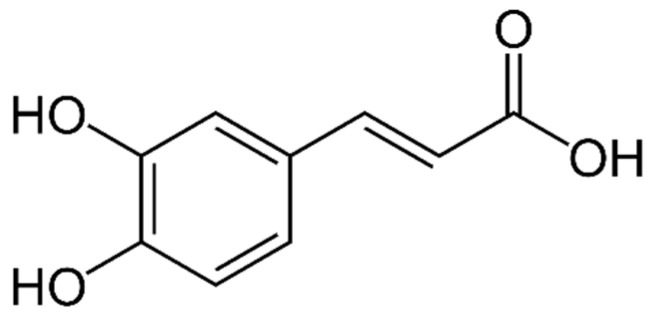
Syringic acid.

**Figure 6:**
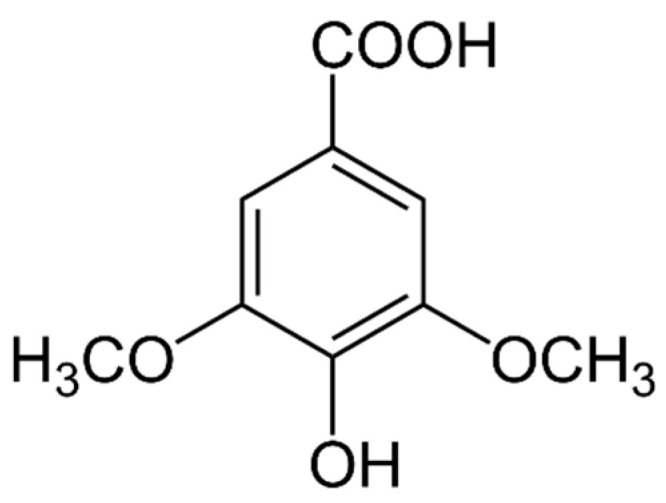
Caffeic acid.

**Figure 7:**
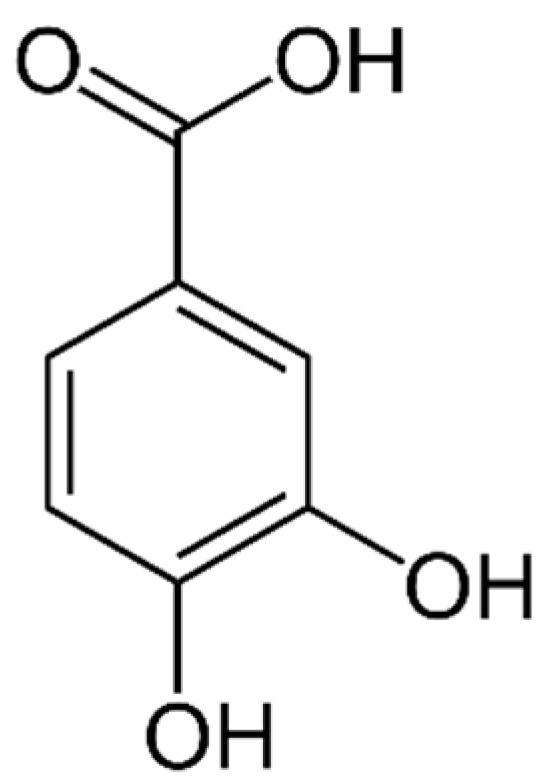
Sinapic acid.

There are two main types of PAs: hydroxybenzoic acids (HBAs) (Figure 2) and hydroxycinnamic acids (HCAs). HBAs include compounds such as gallic acid (Figure 3), protocatechuic acid (Figure 4) and syringic acid (Figure 5), while HCAs include compounds such as caffeic acid (Figure 6), ferulic acid and sinapic acid (Figure 7) [68].

The properties of PAs can vary depending on their structure and the position of the hydroxyl and carboxylic acid groups on the phenolic ring. Some common characteristics of PAs include:

Antioxidant activity: PAs are known to have strong antioxidant activity due to their ability to scavenge free radicals and inhibit lipid peroxidation [140].

Anti-inflammatory activity: PAs have been shown to have anti-inflammatory effects, which may be due to their ability to inhibit the production of inflammatory mediators such as cytokines and prostaglandins [141].

Antimicrobial activity: Some PAs have been shown to have antimicrobial activity against various bacteria and fungi, which may be due to their ability to disrupt microbial cell membranes or inhibit enzyme activity [142].

Absorption and metabolism: PAs are absorbed in the small intestine and metabolized by the liver. The degree of absorption and metabolism can vary depending on the structure of the PA and the presence of other dietary components [143].

#### 4.1.2. Phlorotannins

Phlorotannins, as mentioned earlier, are phenolic compounds that are primarily found in brown algae (Phaeophyceae). Here are some of their characteristics and structures:

Chemical structure: Phlorotannins are phloroglucinol polymers that are formed by the bonding of phloroglucinol units through ether linkages. There are various types of phlorotannins, based on the number of phloroglucinol units they contain and the nature of the linkages between these units [144].

Antioxidant properties: Phlorotannins are known for their strong antioxidant properties, which make them useful in a variety of medical and cosmetic applications [145].

Potential antimicrobial activity: Some studies indicate that phlorotannins may have antimicrobial activity, which could make them useful in the treatment of infections [146].

Potential anti-inflammatory activity: Some studies suggest that phlorotannins may have anti-inflammatory properties, which could make them useful in the treatment of inflammatory conditions [145].

**Figure 8:**
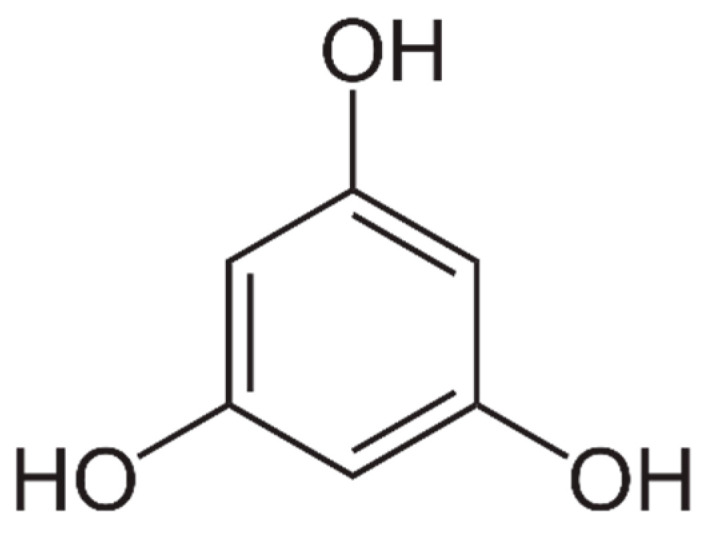
Phloroglucinol.

Phlorotannins are characterized by their complex structure, which typically consists of multiple phloroglucinol units (Figure 8) linked by ether or carbon–carbon bonds. Phlorotannins can vary in size and degree of polymerization, with some larger molecules containing more than 20 phloroglucinol units [144].

#### 4.1.3. Catechins

**Figure 9:**
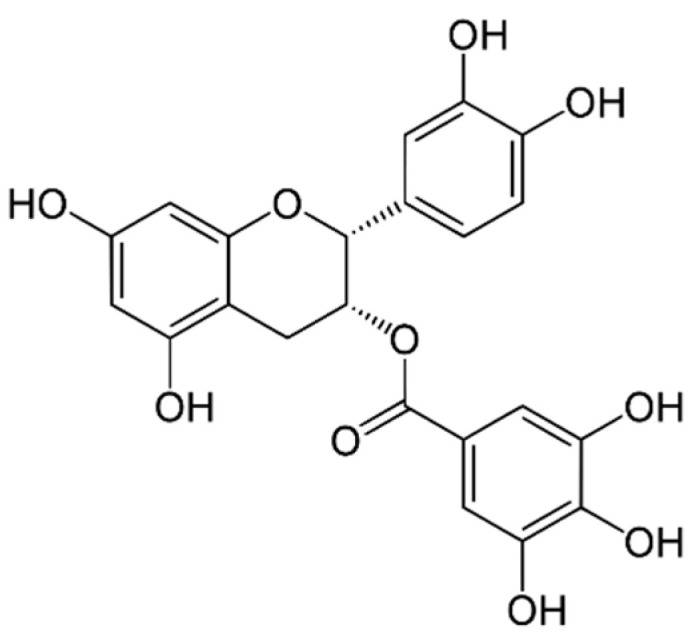
Epicatechin (-3-gallate ECG).

**Figure 10:**
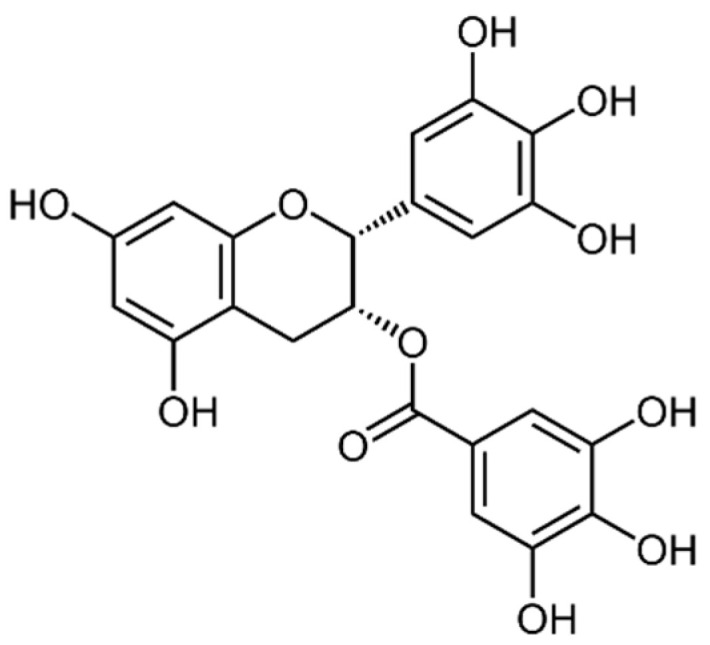
Epigallocatechin-3-gallate (EGCG).

**Figure 11:**
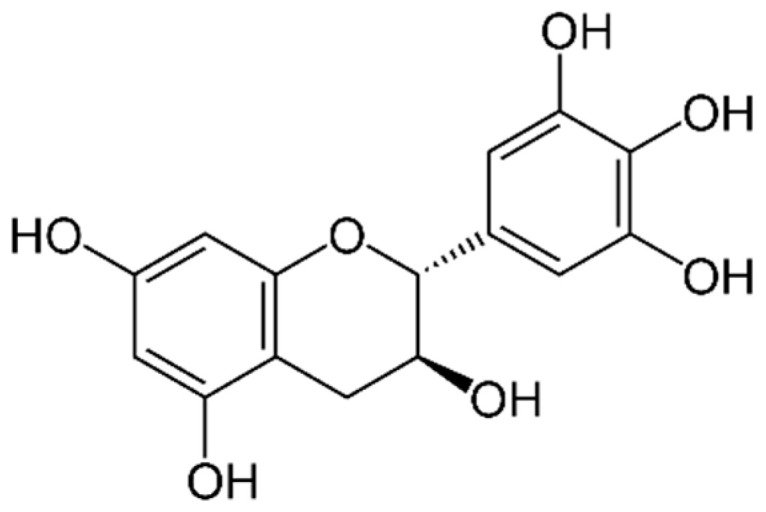
Gallocatechin (GC).

**Figure 12:**
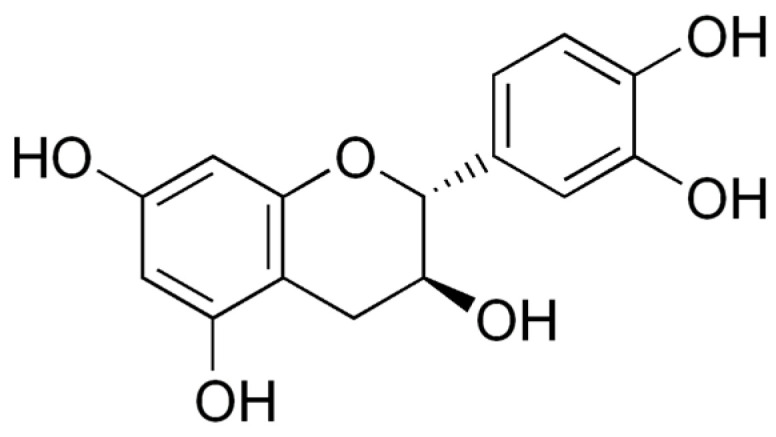
Catechin (C).

Catechins are a type of flavonoid polyphenol found in green tea, but they are also present in some marine algae. They are characterized by a structure that consists of two phenolic rings linked by a carbon–carbon bond, with hydroxyl groups attached to the rings. Catechins can have various substitutions on the rings, which can affect their biological activity [2]. Both rings have hydroxyl groups (-OH) in positions 3 and 4, and in ring B there may be a hydroxyl group in position 5. The position of hydroxyl groups and other substitutions in ring B and ring C can vary, generating different types of catechins with specific biological activities. Some examples of catechins are: epicatechin (EC), epicatechin-3-gallate (ECG) (Figure 9), epigallocatechin (EGC), epigallocatechin-3-gallate (EGCG) (Figure 10), gallocatechin (GC) (Figure 11) and catechin (C) (Figure 12) [147].

Some of the main bioactivities of catechins include:

Antioxidant activity: Catechins have strong antioxidant properties and can scavenge free radicals and reactive oxygen species, which can cause oxidative damage to cells and contribute to various diseases [148]

Anti-inflammatory activity: Catechins have been shown to have anti-inflammatory effects, which may help to reduce the risk of chronic diseases such as cardiovascular disease, diabetes and cancer [149].

Anti-cancer activity: Several studies have suggested that catechins may have anti-cancer properties, particularly in reducing the risk of breast, prostate and colon cancer [150].

Anti-obesity activity: Catechins have been shown to have an anti-obesity effect, particularly by promoting fat oxidation and reducing fat accumulation in the body (2023).

Neuroprotective activity: Catechins have been shown to have neuroprotective effects, which may help to reduce the risk of neurodegenerative diseases such as Alzheimer’s and Parkinson’s disease [151].

Cardiovascular protection: Catechins may help to protect against cardiovascular disease by reducing the risk of hypertension, lowering LDL cholesterol levels and improving endothelial function [152].

Anti-diabetic activity: Catechins may help to regulate blood sugar levels and improve insulin sensitivity, which may be beneficial for people with type 2 diabetes [149].

#### 4.1.4. Bromophenols

**Figure 13:**
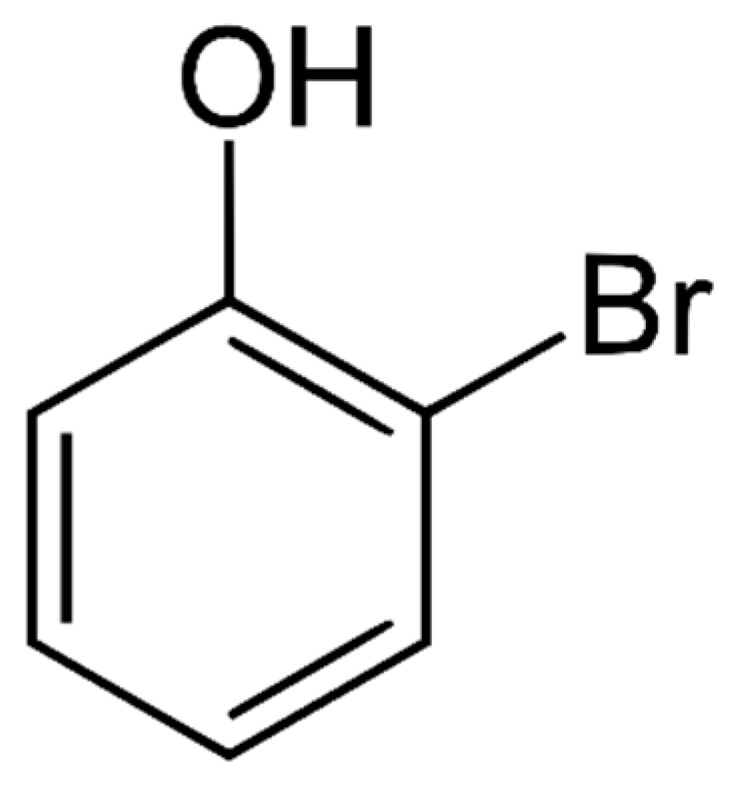
Bromophenol.

Bromophenols (Figure 13) are a type of polyphenol that contain one or more bromine atoms in addition to the phenolic rings. They are found in some marine organisms such as red algae and sponges [153]. Bromophenols can have various structures, with some containing one phenolic ring and others containing two or more rings [23].

Some of the main bioactivities of bromophenols include:

Antioxidant activity: Bromophenols have been shown to have strong antioxidant properties, which can help to protect cells from oxidative damage caused by free radicals and reactive oxygen species [154].

Anti-inflammatory activity: Bromophenols have been shown to have anti-inflammatory effects, which may help to reduce the risk of chronic diseases such as cardiovascular disease, diabetes and cancer [155].

Anti-tumor activity: Several studies have suggested that bromophenols may have anti-tumor properties, particularly in reducing the growth and proliferation of cancer cells [138].

Antibacterial and antiviral activity: Bromophenols have been shown to have antibacterial and antiviral properties, which may help to prevent and treat infections [156].

Neuroprotective activity: Bromophenols have been shown to have neuroprotective effects, which may help to reduce the risk of neurodegenerative diseases such as Alzheimer’s and Parkinson’s disease [157].

Cardiovascular protection: Bromophenols may help to protect against cardiovascular disease by reducing the risk of hypertension, lowering LDL cholesterol levels and improving endothelial function [158].

Anti-diabetic activity: Bromophenols may help to regulate blood sugar levels and improve insulin sensitivity, which may be beneficial for people with type 2 diabetes [159].

#### 4.1.5. Flavonoids

Flavonoids are a diverse class of naturally occurring compounds found in many marine algae, fruits, vegetables and herbs. They are characterized by their unique chemical structure, which consists of two aromatic rings linked by a three-carbon bridge [2]. Flavonoids have a wide range of bioactivities, including:

Antioxidant activity: Flavonoids are well-known for their antioxidant properties, which help to protect cells from oxidative damage caused by free radicals and reactive oxygen species [160].

Anti-inflammatory activity: Many flavonoids have been shown to have anti-inflammatory effects, which may help to reduce the risk of chronic diseases such as cardiovascular disease, diabetes and cancer [161].

Anti-cancer activity: Some flavonoids have been shown to have anti-cancer properties, particularly in reducing the growth and proliferation of cancer cells [162].

Neuroprotective activity: Flavonoids have been shown to have neuroprotective effects, which may help to reduce the risk of neurodegenerative diseases such as Alzheimer’s and Parkinson’s disease [163].

Cardiovascular protection: Flavonoids may help to protect against cardiovascular disease by reducing the risk of hypertension, lowering LDL cholesterol levels and improving endothelial function [164].

Anti-diabetic activity: Flavonoids may help to regulate blood sugar levels and improve insulin sensitivity, which may be beneficial for people with type 2 diabetes [159].

**Figure 14:**
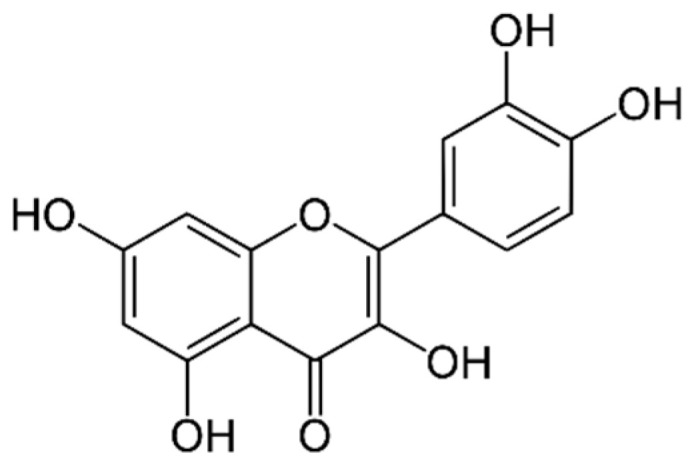
Quercetin.

Some examples of flavonoids and their bioactivities include:

Quercetin (Figure 14): Quercetin is a flavonoid found in many fruits and vegetables, including onions, apples and berries. It has been shown to have antioxidant, anti-inflammatory, anti-cancer and neuroprotective properties [165].

Epigallocatechin gallate (EGCG) (Figure 10): EGCG is a flavonoid found in green tea. It has been shown to have antioxidant, anti-inflammatory, anti-cancer and cardiovascular protective properties [166].

**Figure 15:**
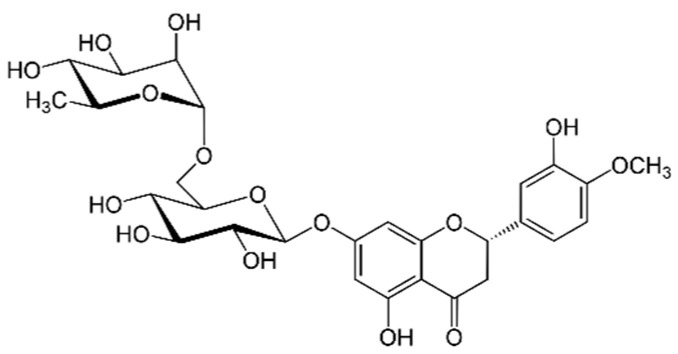
Hesperidin.

Hesperidin (Figure 15): Hesperidin is a flavonoid found in citrus fruits. It has been shown to have antioxidant, anti-inflammatory and cardiovascular protective properties [167].

**Figure 16:**
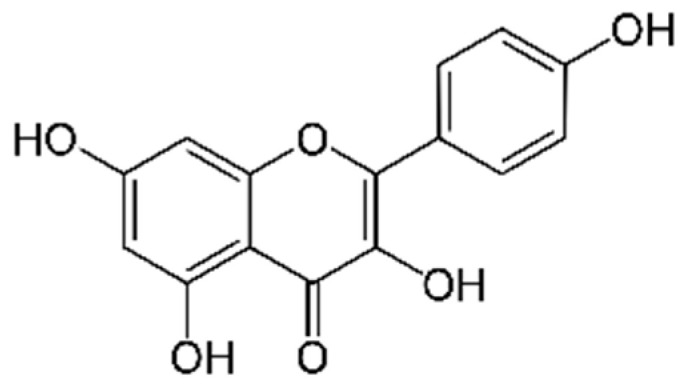
Kaempferol.

Kaempferol (Figure 16): Kaempferol is a flavonoid found in many plants, including broccoli, kale and tea. It has been shown to have antioxidant, anti-inflammatory, anti-cancer and neuroprotective properties [168].

#### 4.1.6. Phenolic Terpenoids

Phenolic terpenoids, also known as terpenophenolics, are a class of natural compounds that consist of a terpenoid backbone (a linear or cyclic hydrocarbon chain) and one or more phenolic groups [19]. They are produced by a wide range of plants and brown and red seaweeds, and they have a diverse array of bioactivities, including:

Antioxidant activity: Phenolic terpenoids are potent antioxidants that can protect cells from oxidative stress caused by free radicals and reactive oxygen species [169].

Anti-inflammatory activity: Many phenolic terpenoids have anti-inflammatory effects, which can help to reduce inflammation in the body and prevent chronic diseases [170].

Anti-cancer activity: Some phenolic terpenoids have been shown to have anti-cancer properties, including inhibiting tumor growth and inducing cancer cell death [171].

Cardiovascular protection: Phenolic terpenoids may help to protect against cardiovascular disease by reducing oxidative stress, inflammation and lipid peroxidation, and improving vascular function [172].

Anti-microbial activity: Some phenolic terpenoids have been shown to have anti-microbial properties, which can help to prevent and treat infections [173].

Neuroprotective activity: Phenolic terpenoids may have neuroprotective effects, including protecting against oxidative damage, reducing inflammation and improving cognitive function [174].

**Figure 17:**
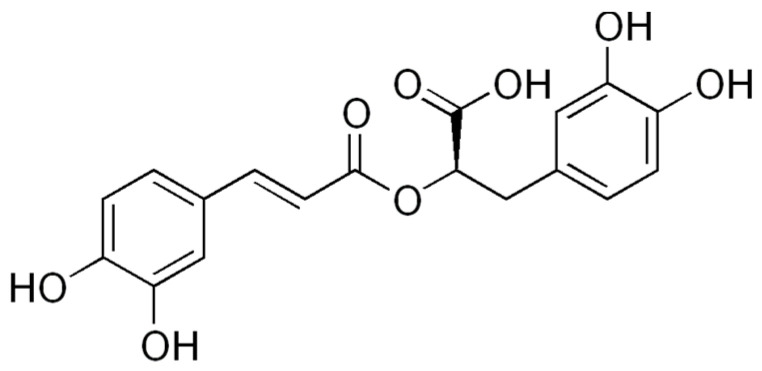
Rosmarinic acid.

Examples of phenolic terpenoids and their bioactivities include:

Rosmarinic acid (Figure 17): Rosmarinic acid is a phenolic terpenoid found in many herbs, including rosemary and sage. It has antioxidant, anti-inflammatory and anti-microbial properties, and may also have neuroprotective effects [175].

**Figure 18:**
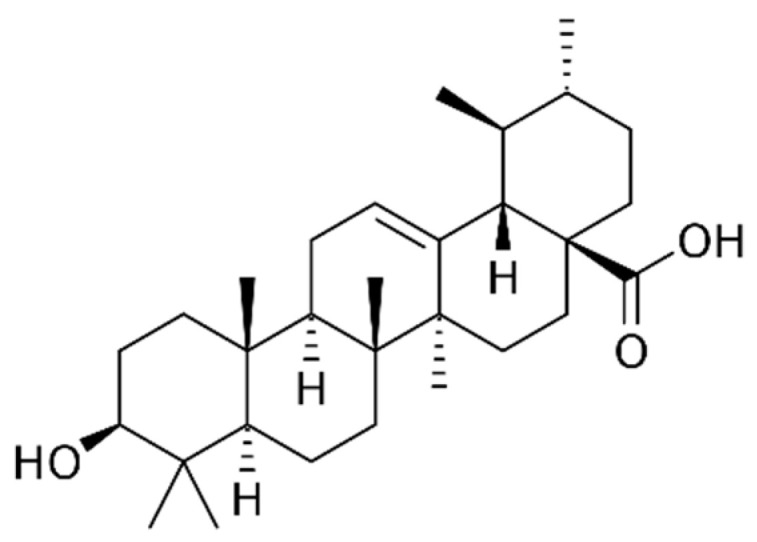
Ursolic acid.

Ursolic acid (Figure 18): Ursolic acid is a pentacyclic triterpenoid found in many fruits and herbs, including apples, rosemary and basil. It has anti-inflammatory, anti-cancer and neuroprotective properties, and may also help to improve cardiovascular health [176].

**Figure 19:**
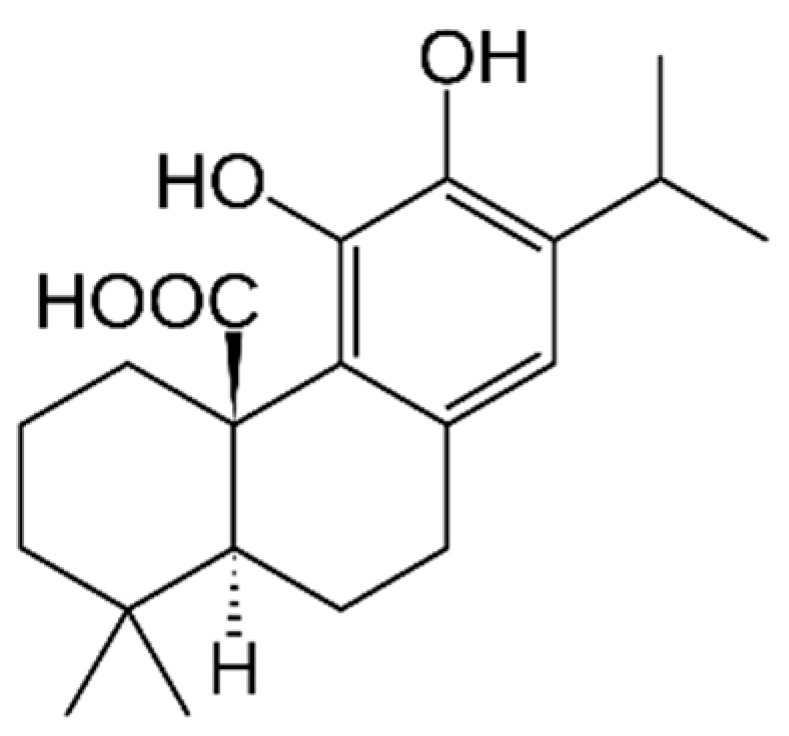
Carnosic acid.

Carnosic acid (Figure 19): Carnosic acid is a phenolic diterpene found in rosemary. It has antioxidant, anti-inflammatory and neuroprotective properties, and may also have anti-cancer effects [177].

**Figure 20:**
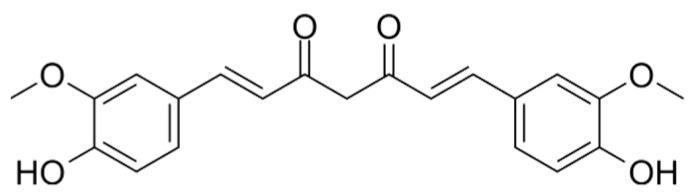
Curcumin.

Curcumin (Figure 20): Curcumin is a polyphenolic terpenoid found in turmeric. It has antioxidant, anti-inflammatory, anti-cancer and neuroprotective properties, and may also help to improve cardiovascular health [178].

Overall, phenolic terpenoids have a wide range of bioactivities that may help to promote health and prevent chronic diseases. However, more research is needed to fully understand the mechanisms of action and potential therapeutic applications of these compounds [179].

#### 4.1.7. Mycosporine-like Amino Acids (MAA)

Mycosporine-like amino acids (MAAs) are a class of water-soluble, low molecular weight compounds that are widely distributed in marine organisms, including cyanobacteria, algae and some invertebrates [180]. They are produced as a response to UV radiation and act as a photoprotective agent, absorbing UV radiation and dissipating it as heat. MAAs have also been found in some marine and terrestrial organisms, including algae, lichens and fungi [181].

**Figure 21:**
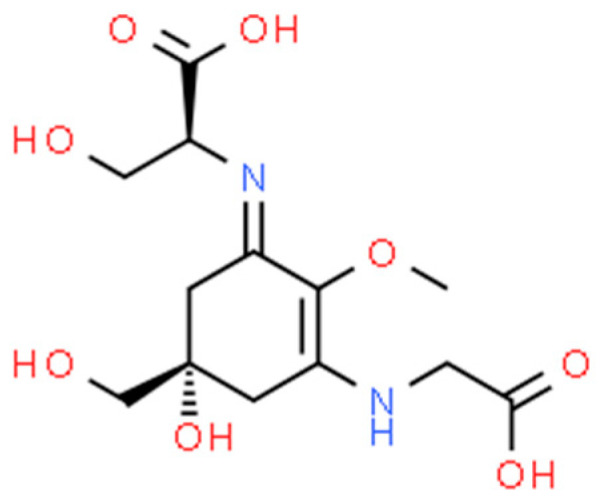
Shinorine.

MAAs have a unique structure that consists of a cyclohexenone or cyclohexenimine chromophore linked to one or more amino acids [134]. The specific structure and number of amino acids can vary depending on the organism and environmental conditions. Some examples of MAAs and their bioactivities include:

Shinorine (Figure 21): Shinorine is an MAA found in red algae. It has been shown to have antioxidant, anti-inflammatory and UV-protective properties [182].

Porphyra-334: Porphyra-334 is an MAA found in red algae (Rhodophyta). It has been shown to have UV-protective properties and may also have anti-inflammatory effects [183].

**Figure 22:**
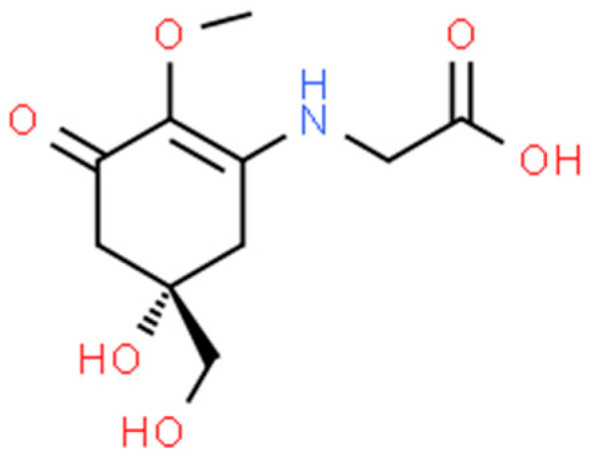
Mycosporine-glycine.

Mycosporine-glycine (Figure 22): Mycosporine-glycine is an MAA found in many marine organisms, including cyanobacteria and algae. It has been shown to have antioxidant and anti-inflammatory properties and may also have neuroprotective effects [184].

**Figure 23:**
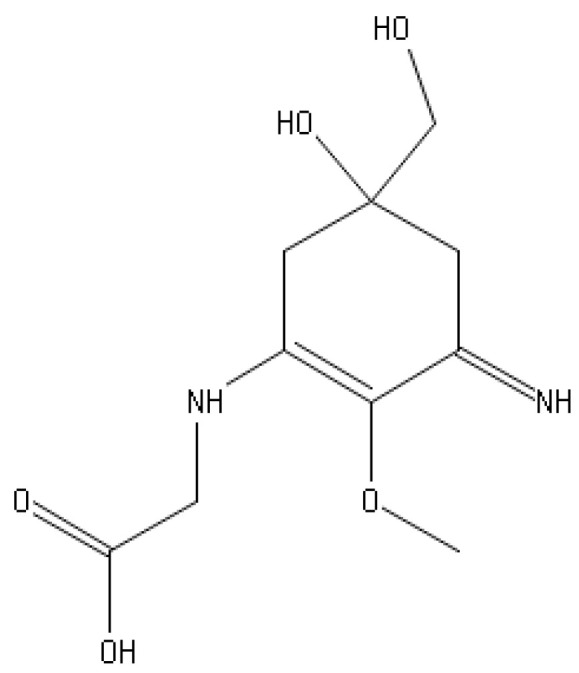
Palythine.

Palythine (Figure 23): Palythine is an MAA found in some invertebrates, including jellyfish and sea anemones. It has been shown to have antioxidant and anti-inflammatory properties and may also have neuroprotective effects [185].

MAAs are known to have several bioactivities, including:

UV-protective activity: MAAs are known for their ability to protect organisms from UV radiation by absorbing UV light and dissipating it as heat. This helps to prevent damage to DNA and other cellular structures caused by UV radiation [181].

Antioxidant activity: MAAs have been shown to have antioxidant properties, which can help to protect cells from oxidative damage caused by free radicals and other reactive oxygen species [186].

Anti-inflammatory activity: Some MAAs have been shown to have anti-inflammatory effects, which may help to reduce inflammation in the body and prevent chronic diseases [187].

Neuroprotective activity: MAAs may have neuroprotective effects, including protecting against oxidative damage and reducing inflammation in the brain [155].

Overall, MAAs are a unique class of compounds with a wide range of bioactivities that are important for the survival of marine organisms in UV-rich environments. More research is needed to fully understand the mechanisms of action and potential therapeutic applications of these compounds [188].

#### 4.1.8. Non-Typical Phenolic Compounds

**Figure 24:**
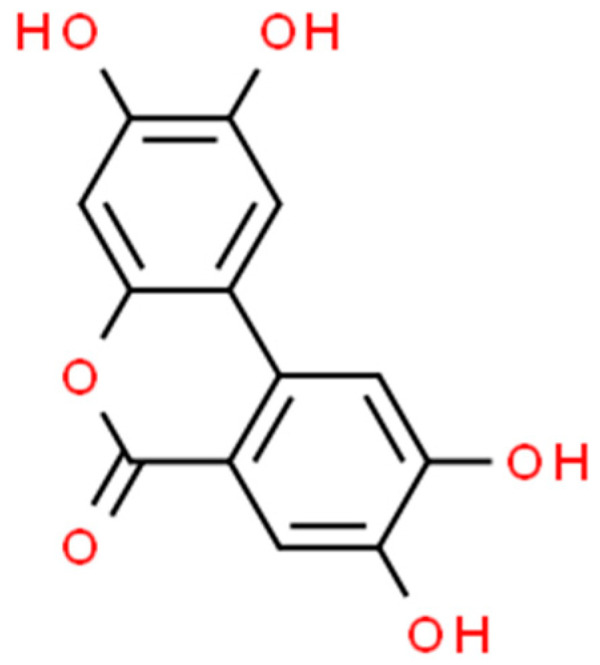
Cladophorol.

Some examples of non-typical phenolic compounds and their bioactivities:

The class of oligomeric polyphenolic compounds known as Cladophorols (Figure 24) were initially discovered and characterized in the green algae *Cladophora socialis* (Chlorophyta) [29]. These compounds have exhibited noteworthy antimicrobial properties, particularly against methicillin-resistant *Staphylococcus aureus* (MRSA). Cladophorol C, a specific compound within this class, has displayed strong selective antibacterial activity against pathogenic MRSA, with a minimum inhibitory concentration (MIC) of 1.4 µg/mL [29].

Several phenolic compounds have been identified in different seaweed species. Colpol, a phenolic compound, has been identified in brown seaweeds, while tichocarpols, a phenylpropanoid derivative, have been identified in the red algae species *Tichocarpus crinitus* (Rhodophyta) [77].

## 5. Phenolic Compound Extraction and Isolation

Pre-treatment with seaweed is advised, such as a washing step to remove stones, sand, epiphytes or other contaminants. As a result, algal biomass can be utilized fresh, dried (air drying or at 30–40 °C with aeration for 3–5 days) or freeze dried [189]. Freeze-dried is preferable because it preserves the integrity of the biomolecules and allows for higher extraction yields [190].

A milling or grinding step is also advised to lower particle size, which would enhance the exposure area between the seaweed biomass and the solvent used for extraction [191]. As a result, the extraction yield will rise.

To avoid co-extraction of pigments or fatty acids [28] with low polar solvents—*n*-hexane, *n*-hexane:acetone, *n*-hexane:ethyl acetate or dichloromethane—a pre-extraction step is usually necessary [19]. The next step is to choose an extraction method, as these approaches vary greatly.

Soxhlet, solid–liquid and liquid–liquid extractions are examples of traditional extraction procedures. Organic solvents (e.g., hexane, petroleum ether, cyclohexane, ethanol, methanol, acetone, benzene, dichloromethane, ethyl acetate, chloroform) are often utilized in the listed techniques. Nonetheless, the solvent used in extraction processes should be non-toxic and inexpensive [192]. Because of its cheaper cost, ethanol is used as an extraction solvent in the industrial sector.

These approaches have changed throughout time to increase extraction efficiency and sustainability as technology has advanced. Currently, ultrasound and microwave-assisted extraction are low-cost, large-scale methods [19].

Following the extraction procedure, the isolated and quantified target phenolic component must be isolated. Depending on the type of substance to be separated, several techniques might be used.

In general, the source of phenolic compounds, the extraction and purification processes used, the sample particle size, the storage conditions and the presence of interfering components in extracts such as fatty acids or pigments all impact the results [19].

Today, phenolic compounds are isolated using preparative chromatography techniques such as column chromatography, high-pressure liquid chromatography (HPLC) or thin-layer chromatography (TLC). However, these chromatographic methods have been developed to be employed for the separation, isolation, purification, identification and quantification of many phenolic substances [193].

Due to these costly procedures, they are still in the initial stage to exploit marine phenolics compounds in an efficient way, although they are being studied to be further applied in pharmaceutics.

## 6. Marine Polyphenols Action Mechanisms

Marine polyphenols are a diverse group of compounds that include flavonoids, phenolic acids and stilbenes, among others. They are synthesized by marine organisms as a defense mechanism against environmental stressors, such as UV radiation, pathogens and predators [3]. Marine polyphenols have been found to exhibit a wide range of biological activities, including anti-inflammatory, anticancer, antiviral, antimicrobial and neuroprotective effects [19].

One of the key mechanisms by which marine polyphenols exert their biological effects is through their ability to interact with cellular signaling pathways. For example, marine polyphenols have been found to modulate the activity of the enzymes involved in cell proliferation, differentiation and apoptosis [194]. This can lead to the inhibition of cancer cell growth and the induction of cell death. Marine polyphenols can also regulate the expression of genes involved in inflammation, such as cytokines and chemokines, thereby reducing inflammation [195].

One of the primary mechanisms of action of marine polyphenols is their ability to scavenge free radicals and reactive oxygen species (ROS) in the body. Free radicals and ROS can damage cells and tissues, leading to inflammation, aging, and chronic diseases. Marine polyphenols have been shown to neutralize free radicals and prevent oxidative stress, thereby protecting cells and tissues from damage [196].

A mechanism by which marine polyphenols exert their effects is through their interaction with cellular membranes. Polyphenols can interact with the lipid bilayer of the membrane, altering its physical properties, such as its fluidity and permeability. This can lead to changes in membrane-associated signaling pathways, affecting cellular functions such as ion transport, receptor activity, and intracellular signaling [197].

Another mechanism of action of marine polyphenols is their ability to modulate the expression of genes and proteins involved in various cellular pathways. For example, marine polyphenols can activate or inhibit enzymes, such as kinases and phosphatases, involved in signal transduction pathways, leading to altered cellular responses. Marine polyphenols can also regulate the expression of transcription factors, such as nuclear factor-kappa B (NF-κB), which plays a critical role in inflammation and immune responses [195,198].

Marine polyphenols can also modulate the gut microbiota, which has important implications for human health. The gut microbiota plays a critical role in nutrient absorption, immune function and metabolic homeostasis [199]. Polyphenols can affect the composition and activity of the gut microbiota, promoting the growth of beneficial bacteria and reducing the growth of harmful bacteria. This can lead to improved gut health and a reduction in the risk of chronic diseases such as inflammatory bowel disease, obesity and type 2 diabetes [200].

Most of the marine phenolic compounds actuated in enzymes, such as cyclooxygenase (COX), work in tandem with nonsteroidal anti-inflammatory medicines (NSAIDs) to suppress the activity or gene expression of pro-inflammatory mediators. Various phenolic compounds can also operate on transcription factors such as nuclear factor-B (NF-B) or nuclear factor-erythroid factor 2-related factor 2 (Nrf-2) to upregulate or downregulate components in antioxidant response pathways. Phenolic chemicals have been utilized to treat a variety of common human disorders, including hypertension, metabolic difficulties, incendiary infections and neurodegenerative diseases, because they can block the enzymes involved in the development of human diseases. Phenolic chemicals have been used to treat hypertension by inhibiting the angiotensin-converting enzyme (ACE). Carbohydrate hydrolyzing enzyme inhibition is a type 2 diabetes mellitus medication, and cholinesterase inhibition is used to treat Alzheimer’s disease [201].

In addition to their biological activities, marine polyphenols have been found to have applications in various industries, such as food, pharmaceuticals and cosmetics [22]. For example, marine polyphenols are used as natural food preservatives due to their antimicrobial activity [202]. They are also used in the development of new drugs and therapies for various diseases, such as cancer and neurodegenerative disorders. Marine polyphenols are also used in the cosmetic industry due to their antioxidant and anti-aging properties [203].

### 6.1. Therapeutic Potential of Marine Polyphenols

#### 6.1.1. Cardiovascular Diseases

Cardiovascular diseases (CVDs) are a leading cause of morbidity and mortality worldwide, and marine polyphenols have been studied extensively for their potential therapeutic effects in CVDs. Some of the ways in which marine polyphenols may be beneficial in CVDs [115] are as follows:

Antioxidant activity: Marine polyphenols have strong antioxidant properties, which can help reduce oxidative stress in the cardiovascular system. Oxidative stress has been implicated in the development and progression of CVDs, and reducing it may help improve cardiovascular health. Some of the main marine polyphenolic compounds with antioxidant activity include [204]:

Phlorotannins: These are a group of complex polyphenolic compounds found in brown seaweeds. Phlorotannins are known for their potent antioxidant activity, and they have been shown to have a wide range of health benefits, including anti-inflammatory and anti-cancer properties [15].

Catechins: These are flavonoid polyphenolic compounds found in green tea and some marine sources, such as seaweed [17]. Catechins have been shown to have potent antioxidant properties, and they may help reduce the risk of cardiovascular disease and other chronic diseases [158].

Flavonoids: These are a group of polyphenolic compounds found in a variety of plant and marine sources. Flavonoids have strong antioxidant properties, and they have been shown to have numerous health benefits, including reducing inflammation, improving cardiovascular health and reducing the risk of certain types of cancer [205].

Phenolic acids: These are a group of polyphenolic compounds found in a variety of marine sources, including marine algae. Phenolic acids have potent antioxidant properties, and they may help reduce the risk of cardiovascular disease and other chronic diseases by reducing oxidative stress [19].

Anti-inflammatory effects: Chronic inflammation is a key factor in the development of CVDs, and marine polyphenols have been shown to possess anti-inflammatory effects. By reducing inflammation, these compounds may help protect against CVDs [206]. Some of the most commonly studied compounds in this regard include:

Fucoidan: This is a sulfated polysaccharide found in brown seaweed and has been shown to possess anti-inflammatory effects by inhibiting the production of pro-inflammatory cytokines [207].

Phlorotannins: These are polyphenolic compounds found in brown seaweed and have been shown to possess anti-inflammatory effects by inhibiting the production of pro-inflammatory enzymes such as cyclooxygenase-2 (COX-2) and inducible nitric oxide synthase (iNOS) [145].

Fucoxanthin: This is a carotenoid pigment found in brown seaweed and has been shown to possess anti-inflammatory effects by inhibiting the production of pro-inflammatory cytokines and reducing oxidative stress [20].

Eckol: This is a phlorotannin found in brown seaweed and has been shown to possess anti-inflammatory effects by inhibiting the production of pro-inflammatory cytokines and reducing oxidative stress [208].

Astaxanthin: This is a carotenoid pigment found in microalgae and has been shown to possess anti-inflammatory effects by inhibiting the production of pro-inflammatory cytokines and reducing oxidative stress [209].

Regulation of lipid metabolism: Dyslipidemia, or abnormal lipid levels in the blood, is a major risk factor for CVDs. Marine polyphenols have been shown to regulate lipid metabolism, potentially reducing the risk of CVDs [210]. Some of the main marine polyphenolic and other minor compounds that have been shown to regulate lipid metabolism and potentially reduce the risk of CVDs are:

Fucoxanthin: This is a carotenoid pigment found in brown seaweed. Fucoxanthin has been shown to reduce body weight, decrease total cholesterol and improve lipid metabolism in animal studies. It works by inhibiting the enzymes involved in the synthesis of cholesterol and triglycerides [211].

Phlorotannins: These are a group of polyphenolic compounds found in brown seaweed. Phlorotannins have been shown to reduce serum lipid levels by inhibiting the absorption of dietary fat and cholesterol. They also exhibit antioxidant and anti-inflammatory properties [15].

Fucoidan: This is a sulfated polysaccharide found in brown seaweed. Fucoidan has been shown to decrease triglyceride levels and improve lipid metabolism in animal studies. It works by inhibiting the activity of the enzymes involved in the synthesis of triglycerides [212].

Astaxanthin: This is a carotenoid pigment found in microalgae, yeast, salmon, trout, krill, shrimp, crayfish, crustaceans and the feathers of some birds. Astaxanthin has been shown to improve lipid metabolism by decreasing serum triglyceride and cholesterol levels. It also exhibits antioxidant and anti-inflammatory properties [213].

Vasodilatory effects: Some marine polyphenols have been shown to have vasodilatory effects, meaning they can help relax blood vessels and improve blood flow. This can help reduce blood pressure and improve cardiovascular health [214]. Some of the main marine polyphenolic and other minor nutrients that have been shown to regulate lipid metabolism and potentially reduce the risk of CVDs are:

Fucoxanthin: This is a carotenoid pigment found in brown seaweed. Fucoxanthin has been shown to reduce body weight, decrease total cholesterol and improve lipid metabolism in animal studies. It works by inhibiting the enzymes involved in the synthesis of cholesterol and triglycerides [215].

Phlorotannins: These are a group of polyphenolic compounds found in brown seaweed. Phlorotannins have been shown to reduce serum lipid levels by inhibiting the absorption of dietary fat and cholesterol. They also exhibit antioxidant and anti-inflammatory properties [216].

Fucoidan: This is a sulfated polysaccharide found in brown seaweed. Fucoidan has been shown to decrease triglyceride levels and improve lipid metabolism in animal studies. It works by inhibiting the activity of the enzymes involved in the synthesis of triglycerides [217].

Platelet inhibition: Platelet activation and aggregation play a key role in the development of thrombosis, which can lead to heart attacks and strokes. Marine polyphenols and other minor nutrients have been shown to inhibit platelet aggregation, potentially reducing the risk of thrombosis [218]. Some of the main ones are:

Fucoidan: Fucoidan is a sulfated polysaccharide found in various types of brown seaweed. It has been shown to inhibit platelet aggregation by inhibiting the binding of platelet activating factors to platelet receptors [219].

Phlorotannins: Phlorotannins have been shown to inhibit platelet aggregation by interfering with the release of platelet activating factors [220].

Catechins: Catechins, a type of flavonoid found in many types of seaweed, can inhibit platelet aggregation by inhibiting the activity of platelet-activating factors and reducing the adhesion of platelets to the blood vessel wall [221,222].

Eckol: Eckol is a type of phlorotannin found in brown seaweeds. It has been shown to inhibit platelet aggregation by interfering with the binding of platelet activating factors to platelet receptors [223].

#### 6.1.2. Diabetes

Among the marine polyphenols that have been studied for their potential therapeutic effects in diabetes (Table 2), some of the most commonly studied include:

Fucoxanthin: This polyphenol has been shown to have anti-diabetic effects by improving insulin sensitivity and glucose metabolism in animal studies [224].

Phlorotannins: These polyphenols have been shown to have anti-diabetic effects by reducing blood glucose levels and improving insulin sensitivity in animal studies [225].

Fucoidan: This polysaccharide has been shown to have anti-diabetic effects by improving glucose metabolism and insulin sensitivity in animal studies [6].

Bromophenols: These polyphenols have been shown to have anti-diabetic effects by reducing blood glucose levels and improving insulin sensitivity in animal studies [226].

Catechins: These polyphenols have been shown to have anti-diabetic effects by improving insulin sensitivity and glucose metabolism in animal studies.

While these marine polyphenols have shown promising potential in animal studies, further research is needed to determine their efficacy and safety in humans before they can be recommended as a therapeutic option for diabetes [227].

**Table 2:** Therapeutic potential of marine polyphenols for Diabetes.

Seaweed	Compound	Animal/Cell Line	Effect	Reference
*E. cava*	fucodiphloroethol G, dieckol, 6,6′-bieckol, 7-phloroeckol, phlorofucofuroeckol-A	In vitro assay: α-glucosidase and α-amylase inhibitory activity	Inhibition of α-glucosidase (IC_50_ values ranged from 10.8 μM for dieckol to 49.5 μM for 7-phloroeckol) and α-amylase (IC_50_ values ranged from 125 μM for dieckol to <500 μM for the rest of compounds, except 7-phloroeckol with a value of 250 μM) activities	[228]
*Lessonia trabeculate*	Polyphenol-rich extracts	In vitro assay: α-glucosidase and lipase activity	Inhibition of α-glucosidase and lipase activities (IC_50_ < 0.25 mg/mL)	[229]
*F. vesiculosus*	Crude extract and semi-purified phlorotannins composed by fucols, fucophlorethols, fuhalols and several other phlorotannin derivatives	In vitro assay: α-glucosidase, α-amylase and pancreatic lipase inhibitory activity	Inhibition of α-amylase (IC_50_~28.8–2.8 μg/mL), α-glucosidase (IC_50_~4.5–0.82 μg/mL) and pancreatic lipase (IC_50_~45.9–19.0 μg/mL) activities	[230]
*Rhodomela confervoides*	3,4-dibromo-5-(2-bromo-3,4-dihydroxy-6-(ethoxymethyl)benzyl)benzene-1,2-diol)	In vitro: insulin resistant C2C12 cells treated with bromophenol (0.1–0.5 μM for phenol)	Inhibition of PTP1B activity (IC_50_~0.84 μM) Activation of insulin signaling and potentiate insulin sensitivity	[231]
*Rhodomela confervoides*	3-Bromo-4,5-bis(2,3-dibromo-4,5-dihydroxybenzyl)-1,2-benzenediol	In vitro: palmitate-induced insulin resistance in C2C12 cells treated with bromophenol (0.5–2.0 μM for phenol)	Inhibition of PTP1B activity (IC_50_~2 μM) Activation of insulin signaling and prevent palmitate-induced insulin resistance	[232]
*E. stolonifera*	Phlorofucofuroeckol-A	In vitro assay for non-enzymatic insulin glycation	Inhibition of AGEs formation (IC_50_ 29.50–43.55 μM for D-ribose and D-glucose-induced insulin glycation, respectively)	[233]
*Ishige foliacea*	Octaphlorethol A	In vitro: STZ-induced pancreatic β-cell damage (RINm5F pancreatic β-cells) (12.5–50.0 μg/mL for phenol)	Decreased the death of STZ-treated pancreatic β-cells Decreased the TBARS and ROS Increased the activity of antioxidant enzymes	[234]
*E. cava*	6,6-Bieckol, phloroeckol, dieckol and phlorofucofuroeckol	In vivo: high glucose-stimulated oxidative stress in zebrafish, a vertebrate model (10–20 μM of phenols)	Inhibition of high glucose-induced ROS and cell death Dieckol reduced the heart rates, ROS, NO and lipid peroxidation Dieckol reduced the overexpression of iNOS and COX-2	[235]
*Ulva prolifera*	Extract rich in flavonoids	In vivo: STZ-induced diabetic rats (150 mg/kg/day bw of phenol for 4 weeks by gavage)	Diminished the fasting blood glucose and improved oral glucose tolerance Hypoglycemic effect by increasing IRS1/PI3K/Akt and suppressing JNK1/2 in liver	[236]

#### 6.1.3. Neurodegenerative Diseases

Neurodegenerative diseases are a group of chronic and progressive disorders that affect the nervous system and lead to the gradual loss of function of neurons. They include Alzheimer’s disease, Parkinson’s disease and Huntington’s disease, among others. The pathogenesis of these diseases is multifactorial and involves oxidative stress, inflammation and the accumulation of misfolded proteins [237].

Marine polyphenols are natural compounds found in various marine organisms, including seaweeds (Table 3), algae and marine animals. They have been shown to possess a wide range of biological activities, including antioxidant, anti-inflammatory and neuroprotective effects. Therefore, marine polyphenols have been investigated for their therapeutic potential in the prevention and treatment of neurodegenerative diseases [9].

The antioxidant properties of marine polyphenols can help reduce oxidative stress in neurons, which is a major contributor to neurodegeneration [238]. These compounds have been shown to scavenge free radicals, prevent lipid peroxidation and enhance the activity of antioxidant enzymes. Moreover, marine polyphenols can also modulate inflammatory pathways, reducing the release of pro-inflammatory cytokines and chemokines that contribute to neuronal damage [2].

Marine polyphenols have also been found to have neuroprotective effects by inhibiting the aggregation of misfolded proteins, such as amyloid-beta and tau in Alzheimer’s disease and alpha-synuclein in Parkinson’s disease. By preventing the accumulation of these proteins, marine polyphenols can help maintain neuronal function and prevent neuronal death [239].

Overall, the therapeutic potential of marine polyphenols in neurodegenerative diseases is promising, but more research is needed to fully understand their mechanisms of action and to develop effective treatments. Further studies should focus on identifying the most potent marine polyphenols and optimizing their delivery to the brain to maximize their therapeutic effects [240].

Phenolic compounds and other minor nutrients from marine sources have shown potential in the treatment of neurodegenerative diseases due to their antioxidant and anti-inflammatory properties [241]. Some of the main phenolic compounds of marine origin with potential in the treatment of neurodegenerative diseases include:

Phlorotannins: These are a type of polyphenol found in brown seaweed that have been shown to have neuroprotective effects. They have been shown to reduce oxidative stress and inflammation in the brain, which are two factors that contribute to neurodegeneration [242].

Fucoxanthin: This is a carotenoid pigment found in brown seaweed that has been shown to have anti-inflammatory and antioxidant properties. It has been shown to reduce inflammation in the brain and to protect against oxidative stress [243].

Fucoidan: This is a sulfated polysaccharide found in brown seaweed that has been shown to have neuroprotective effects. It has been shown to reduce inflammation in the brain and to protect against oxidative stress [244].

Halogenated phenols: These are phenolic compounds that are found in marine sponges and have been shown to have neuroprotective effects. They have been shown to protect against oxidative stress and to reduce inflammation in the brain [3].

Bromophenols: These are phenolic compounds that are found in marine algae and have been shown to have neuroprotective effects. They have been shown to protect against oxidative stress and to reduce inflammation in the brain [245]. 

**Table 3:** Therapeutic potential of marine polyphenols for neurodegenerative diseases.

Seaweed	Compound	Animal/Cell Line	Effect	Reference
*E. cava*	dieckol, 6,6′-bieckol, 8,8′-bieckol, eckol and phlorofucofuroeckol-A	In vitro: assays of AChE, BChE and BACE-1 activities -- In vitro: Jurkat clone E1–6 cells (GSK3β activity at 50 μM)	Inhibition of AChE and BChE activities (IC_50_ 16.0–96.3 μM and 0.9–29.0 μM, respectively) Inhibition of BACE-1 activity (18.6–58.3% at 1 μM) Inhibition of GSK3β activity (14.4–39.7% at 50 μM)	[246]
*E. bicyclis*	eckols	In vitro: assays of AChE and BChE activities	Inhibition of AChE and BChE activities (IC_50_ 2.78 and 3.48 μg/mL, respectively)	[247]
*Gracilaria beckeri*, *Gelidium pristoides*, *U. rigida* and *E. maxima*	Aqueous extracts composed by phloroglucinol, catechin and epicatechin 3-glucoside	In vitro: assays of AChE and BChE activities	High antioxidant potency Inhibition of AChE and BChE activities (IC_50_ 49.41 and 52.11 μg/mL, respectively, for *E. maxima*) Inhibition of Aβ aggregation	[248]
*E. maxima*, *G. pristoides*, *Gracilaria gracilis* and *Ulva lactuca*	Aqueous-ethanolic extracts containing phlorotannins, flavonoids and phenolic acids	In vitro: assays of AChE, BChE and BACE-1 activities	Inhibition of AChE and BChE activities (IC_50_ 1.74–2.42 and 1.55–2.04 mg/mL, respectively) Inhibition of BACE-1 activity (IC_50_ 0.052–0.062 mg/mL) Inhibition of Aβ aggregation	[249]
*E. cava*	Phlorofucofuroeckol	In vitro: Glutamate-stimulated PC12 cells (10 μM of phenol)	Increased the cell viability and attenuated glutamate excitotoxicity Inhibited the apoptosis in a caspase-dependent manner Regulated the production of ROS and attenuated mitochondrial dysfunction	[250]
*E. cava*	Phloroglucinol	In vitro: Aβ-induced neurotoxicity in HT-22 cells (10 μg/mL) --- In vivo: 5XFAD mice, model of AD (acute, 1.2 μmol of phenol bilaterally delivery)	Reduced the Aβ-induced ROS accumulation in HT-22 cells Ameliorated the reduction in dendritic spine density --- Attenuated the impairments in cognitive dysfunction	[251]
*E. maxima*	Eckmaxol	In vitro: Aβ oligomer-induced neurotoxicity in SH-SY5Y cells (5–20 μM of phenol)	Prevented the Aβ oligomer-induced neurotoxicity Inhibition of GSK3β and ERK signaling pathway	[252]
*E. cava*	eckol, 8,80-bieckol and dieckol	In vitro: Aβ 25–35-induced damage in PC12 Cells (1–50 μM of phenol)	Inhibition of pro-inflammatory enzymes preventing Aβ production and neurotoxicity on the brain	[253]
*E. cava*	dieckol, 6,6′-bieckol, 8,8′-bieckol, eckol and phlorofucofuroeckol-A	In vitro: assays of AChE, BChE and BACE-1 activities -- In vitro: Jurkat clone E1–6 cells (GSK3β activity at 50 μM)	Inhibition of AChE and BChE activities (IC_50_ 16.0–96.3 μM and 0.9–29.0 μM, respectively) Inhibition of BACE-1 activity (18.6–58.3% at 1 μM) Inhibition of GSK3β activity (14.4–39.7% at 50 μM)	[246]
*E. bicyclis*	eckols	In vitro: assays of AChE and BChE activities	Inhibition of AChE and BChE activities (IC_50_ 2.78 and 3.48 μg/mL, respectively)	[247]
*Gracilaria beckeri*, *Gelidium pristoides*, *U. rigida* and *E. maxima*	Aqueous extracts composed by phloroglucinol, catechin and epicatechin 3-glucoside	In vitro: assays of AChE and BChE activities	High antioxidant potency Inhibition of AChE and BChE activities (IC_50_ 49.41 and 52.11 μg/mL, respectively, for *E. maxima*) Inhibition of Aβ aggregation	[248]

#### 6.1.4. Cancer

As described earlier, polyphenols (Table 4) and other micronutrients are bioactive compounds found in plants and animals, and recently there has been a growing interest in marine polyphenols due to their therapeutic potential in several areas of health, including cancer [2,254].

Marine polyphenols are extracted from marine organisms such as algae, mollusks, corals, sponges and fish. They have a wide variety of health benefits, including antioxidant, anti-inflammatory, anticancer and immunomodulatory activities [255].

The anticancer activity of marine polyphenols has been observed in several in vitro and in vivo studies. They are able to induce cell death in cancer cells, inhibit cell proliferation, inhibit angiogenesis and modulate the immune response. These effects are important because uncontrolled cell proliferation, excessive angiogenesis and suppression of the immune response are hallmarks of tumor development [256].

Ellagic acid is a polyphenol present in kelp that has been shown to cause cell death in breast and colorectal cancer. It functions by blocking the expression of pro-inflammatory and pro-angiogenic genes in cancer cells. It has also been shown to boost the production of tumor suppressor proteins [257].

Phloroglucinol acid is another polyphenol found in marine sponges with anticancer activity against lung and prostate cancer cells. This polyphenol induces apoptosis (programmed cell death) in cancer cells and inhibits the formation of capillaries that are necessary for angiogenesis [138].

Another micronutrient of marine origin with therapeutic potential is fucoidan, a sulfated polysaccharide found in brown algae. Studies suggest that fucoidan has anticancer activity against several cancer cell lines, including breast, lung and colon cancer cells. This sulfated polysaccharide inhibits angiogenesis, modulates the immune response and induces apoptosis in cancer cells [258].

Fucoxanthin is a carotenoid pigment found in brown algae that has also been shown to have anticancer activity. This compound is capable of inhibiting the growth of liver and colon cancer cells, inhibiting cell proliferation and inducing apoptosis [211].

The eckol-family of phlorotannins stands out among the various phlorotannin structures due to its exceptional bioactivity, particularly its anti-tumoral properties [254].

Despite the therapeutic potential of marine polyphenols in cancer, more research is needed to fully understand their mechanisms of action and to develop new anticancer therapies based on these compounds. Furthermore, it is important to evaluate the safety and efficacy of these compounds in human clinical trials [259].

**Table 4:** Therapeutic potential of marine polyphenols for cancer.

Specie	Compound	Animal/Cell Line	Effect	Reference
*E. bicyclis*	Phlorofucofuroeckol A	In vitro: LoVo, HT-29, SW480 and HCT116 cells (25–100 μM of phenol)	Antiproliferative and pro-apoptotic properties Induced the apoptosis on colorectal cancer cells by ATF3 signalling pathway	[260]
*E. cava*	Phloroglucinol	In vitro: MCF7, SKBR3 and BT549 cells (10–100 μM of phenol) In vivo: MDA-MB231 breast cancer cells implanted into mammary fat pads of NOD-scid gamma (NSG) mice, treated with phloroglucinol 4 times on alternate days (25 mg/kg bw by intratumoral injections)	Antiproliferative effect by KRAS inhibition and its downstream PI3K/Akt and RAF-1/ERK signalling pathways	[261]
*E. cava*	Dieckol	In vivo: *N*-nitrosodiethylamime-induced hepatocarcinogenesis rats (40 mg/kg bw/day for 15 weeks administered orally)	Regulated the xenobiotic-metabolizing enzymes Induced the apoptosis by mitochondrial pathway Inhibited the invasion by decreasing PCNA expression Inhibited the angiogenesis by changing MMP-2 and MMP-9 activity and VEGF expression Anti-inflammatory activity by inhibiting NF-kB and COX2	[262]
*E. cava*	Dieckol	In vitro: EA.hy926 cells (10–100 μM of phenol)	Antiangiogenic activity by inhibiting the proliferation and migration of cells through MAPK, ERK and p38 signaling pathways	[263]
*E. cava*	Eckol	In vitro: on human HaCaT keratinocytes against PM2.5-induced cell damage (30 μM of phenol for 17 days)	Decreased ROS generation Protected the cells from apoptosis by inhibiting MAPK signaling pathway	[264]
*E. cava*	Dieckol	In vivo: *N*-nitrosodiethylamime-induced hepatocarcinogenesis rats (40 mg/kg bw/day for 15 weeks administered orally)	Regulated the xenobiotic-metabolizing enzymes Induced the apoptosis by mitochondrial pathway Inhibited the invasion by decreasing PCNA expression Inhibited the angiogenesis by changing MMP-2 and MMP-9 activity and VEGF expression Anti-inflammatory activity by inhibiting NF-kB and COX2	[262]

## 7. Safety and Toxicity of Marine Polyphenols

As previously stated, marine polyphenols are natural substances found in a variety of aquatic creatures, including seaweed, algae and shellfish. These compounds have received a significant amount of attention because of their possible health advantages, which include antioxidant, anti-inflammatory and anti-cancer properties. However, concerns have been raised regarding their safety and toxicity [9,18], mostly regarding their extraction and isolation methods, which can change their relative safety and toxicity; due to the diverse chemical structure and impurities, there is a need to standardize the procedure from extraction until the safety/toxicity assays.

Several studies have investigated the safety of marine polyphenols and their potential toxicity. Overall, the available evidence suggests that these compounds are generally safe for human consumption. However, there are some concerns regarding their potential toxicity at high doses [19]. To date, the bioavailability of seaweeds has not been well researched. More research and study are required in this sector. The majority of seaweed phenolic pharmacological and biological bioavailability investigations have used mice models. Animal investigations and in vitro studies have provided evidence that seaweed phenols protect against various illnesses. As a result, fresh research investigations are required to investigate and completely comprehend their bioavailability in humans (the proportion of the chemical that reaches the human circulatory system and has an active impact). Furthermore, there is more pharmacokinetics required in order to fully understand the marine phenolic potential in in vivo models, due to a general lack of information, where the therapeutics in use do not have full public data regarding this topic [19,201,265,266]. Although phenolic compounds appear to have a wide range of biological actions, the problem of safe dose must be addressed. Indeed, phenolic substances have bimodal pharmacological effects, in that they can be beneficial at low doses while being poisonous at large concentrations. However, much current research on the negative effects of phenolic chemicals is focused on cell investigations and animal models, with few human trials [266].

One of the main concerns is the potential for heavy metal contamination in marine organisms. Heavy metals such as lead, mercury and cadmium can accumulate in the tissues of marine organisms and can pose a health risk if consumed in large quantities. Therefore, it is essential to ensure that marine polyphenol supplements are sourced from reputable suppliers that test for heavy metal contamination [267].

Another concern is the potential for allergic reactions to marine polyphenols. Some people may be allergic to certain types of marine organisms or their products, which could lead to adverse reactions. Therefore, it is important to check for any allergies before consuming marine polyphenol supplements [268].

Furthermore, the effects of marine polyphenols on pregnant and breastfeeding women are not yet fully understood, and caution should be exercised when consuming these compounds during these periods [269].

In conclusion, marine polyphenols have shown potential health benefits, but it is essential to ensure their safety and minimize any potential toxicity. It is recommended to consume marine polyphenols in moderation and to obtain them from reputable sources. Furthermore, consulting with a healthcare expert before beginning any new supplement routine is always recommended [270].

### Phenolic Compound Pharmacodynamics

Pharmacodynamics and pharmacokinetics depend on the bioavailability of themarine phenolic compounds and are conducted by the absorptive process across the intestine into the circulatory system, after food ingestion. Thus, bioavailability involves several processes, including liberation from a food matrix, absorption, distribution, metabolism and elimination phases. Several polyphenols can be ingested as either purified, isolated substances or in foods. During the absorption process, gastric acid from the stomach can cause initial modifications to oligomeric polyphenols. Following ingestion, glycosidic polyphenols are cleaved in the small intestine, releasing the glycoside radical. Lactase phlorizin hydrolase and cytosolic glucosidase are enzymes with an affinity for glucose, xylose and galactose. However, polyphenols that are not cleaved by these enzymes are not absorbed by the small intestine and can be cleaved into small molecules known as phenolic acids produced by intestinal bacteria. Polyphenol structures can also be involved in conjugation reactions, resulting in methyl, glucuronide or sulfate groups. The remaining polyphenols, especially those attached to rhamnose, can be processed by rhamnosidase released by the colonic microbiota. Following these absorptive processes, phenolics will typically follow one of four paths: (1) Excretion in the feces; (2) absorption by the mucosa of the intestines or the colon, followed by entry into the portal vein for delivery to the liver; (3) further conjugation in the liver can result in the addition of with methyl, glucuronide or sulfate groups, followed by release into the bloodstream for tissue absorption; and (4) excretion in the urine. However, the absorption kinetic is mostly determined by the physical and chemical properties of the bioactive substances, but it can also be impacted by the subject’s physiology (age, genetic profile, gender, lifestyle, etc.), resulting in a unique bioavailability profile. As a result, the half-life of bioactive chemicals can range from minutes (e.g., gallic acid) to hours (e.g., rutin) [201,266].

## 8. Challenges and Opportunities in the Use of Marine Polyphenols as a Therapy

Due to these activities, there has been increasing interest in using marine polyphenols as a therapy for various diseases. However, the use of marine polyphenols as a therapy also presents several challenges. One of the challenges is the identification and isolation of specific marine polyphenols with therapeutic potential [9,18]. Marine organisms contain a vast array of compounds, and it can be challenging to isolate specific polyphenols with therapeutic potential. Additionally, there is a general absence of standardized techniques for the extraction and purification of marine polyphenols, which can affect the quality and consistency of the final product [12].

Another challenge is the limited knowledge of the pharmacokinetics and pharmacodynamics of marine polyphenols. Unlike synthetic drugs, marine polyphenols have complex structures that can affect their bioavailability, absorption, distribution, metabolism and excretion. This complexity can make it challenging to determine the optimal dose and frequency of administration of marine polyphenols [271].

Despite these challenges, there are several opportunities in the use of marine polyphenols as a therapy. One of the opportunities is the development of new therapies for diseases that currently have limited treatment options. For example, marine polyphenols have shown promising results as a therapy for diverse types of cancer, including breast, colon and prostate cancer. They have also shown potential as a therapy for neurodegenerative diseases such as Alzheimer’s and Parkinson’s [2].

Another opportunity is the development of new products for the food and cosmetic industries. Marine polyphenols have been shown to possess anti-aging and skin-whitening properties, making them attractive ingredients for the cosmetic industry [22]. Additionally, marine polyphenols have been shown to have antimicrobial properties, making them potential additives for food preservation [202].

Therefore, the use of marine polyphenols as a therapy presents both challenges and opportunities. Despite the challenges, the potential benefits of marine polyphenols in the treatment of various diseases and the development of new products make them a promising area of research. Further studies are needed to address the challenges and fully exploit the opportunities in the use of marine polyphenols as a therapy [18].

However, to obtain the benefits of phenolic compounds’ biological activities, they must be consumed. These chemicals have traditionally been included directly into meals, but their instability during food processing, distribution and storage, as well as their limited absorption and bioavailability in the gastrointestinal system, restrict their activity and health effects. Similarly, topical polyphenol usage is restricted due to their rapid oxidation, which causes food browning and the creation of undesirable aromas, as well as a decrease in activity [266,272]. Their encapsulation or application as typical drugs on a delivery system can potentiate their bioactivity and respective benefits [265,272,273].

### Approved Polyphenolic Therapeutics

The most explored seaweed components are phenolic compounds, which are currently used in commercial solutions (for example, cosmetic items). Normally, phenolic compounds are not separated because commercial seaweed extracts include a high concentration of phenols [19].

The European Food Safety Authority has certified SeapolynolTM (Botamedi Inc, Seoul, Korea) as a food supplement. This supplement is based on dieckol and other polyphenols derived from *E. cava*; it has been evaluated and shown to be effective as an anti-hyperlipidemic and cardioprotective agent against doxorubicin-induced cardiotoxicity. Furthermore, SeapolynolTM improved insulin sensitivity in type 2 diabetes and may play an important role in the prevention of metabolic diseases [19,274,275,276,277] These tests, however, were carried out only on mice. The primary goals of phlorotannin supplements in cardiovascular illness are to avoid arteriosclerosis and enhance protective high-density lipoprotein cholesterol (HDL-C). HealSeaTM (made by Diana Naturals in Rennes, France), IdAlgTM (manufactured by Bio Serae in Bram, France) and SeanolTM (produced by LiveChem in Jeju-do, South Korea and sold by Simple Health in Maitland, USA) are phlorotannin-containing products [19,278]. InSea2TM (Rimouski, QC, Canada), a commercial combination of *A. nodosum* and *F. vesiculosus* phlorotannins, promotes a 90% decrease in postprandial blood glucose while lowering peak insulin production by 40% [19].

Among the sea urchin pigments, a cardiovascular drug, Histochrome (solution of Echinochrome A sodium salt), is the only compound that has been approved for clinical use. It is used in solutions of 10 mg/mL for cardiology and 2 mg/mL for ophthalmology. It is recommended for clinical use in patients with acute myocardial infarction to decrease incidence of ventricular extra systole and episodes of accelerated idioventricular rhythm after thrombolytic therapy [133].

## 9. Conclusions and Future Perspectives

The therapeutic potential of polyphenols and other micronutrients of marine origin has acquired significant attention in recent years due to their numerous health benefits. Polyphenols are a class of natural compounds found in several marine organisms that have antioxidant and anti-inflammatory properties. Other micronutrients found in marine sources include omega-3 fatty acids, vitamins and minerals, all of which have been linked to various health benefits [2].

Polyphenols and other micronutrients of marine origin have been shown in studies to help avoid and cure a variety of health problems, including cardiovascular disease, cancer, diabetes and neurodegenerative illnesses. Omega-3 fatty acids, for example, have been shown to promote cardiac health by decreasing inflammation, improving blood lipid levels, and lowering blood pressure. Polyphenols have also been shown to have anti-cancer properties by inhibiting the growth of cancer cells and promoting their death [279].

In addition to their therapeutic potential, marine-based polyphenols and micronutrients are also being investigated for their potential use in cosmetic and skincare products. Studies have shown that marine-derived compounds can have a helpful influence on skin health by decreasing inflammation, improving collagen production and protecting against UV damage [22].

Despite the promising potential of marine-based polyphenols and other micronutrients, there is still much research to be done to completely identify their mechanisms of action and potential side effects. However, the growing body of evidence suggests that marine-based compounds have significant therapeutic potential and should be further investigated for their potential use in disease prevention and treatment [13,254].

In conclusion, the therapeutic potential of marine-based polyphenols and other micronutrients is a promising area of research that has the potential to significantly impact human health [2]. Ongoing research into the mechanisms of action and potential side effects of these compounds will provide valuable insights into their therapeutic potential and pave the way for the development of new therapies and preventive measures. As such, marine-based compounds should be further investigated as a valuable resource for disease prevention and treatment [280].

## Data Availability

Not applicable.
